# Genome wide association study of seedling and adult plant leaf rust resistance in two subsets of barley genetic resources

**DOI:** 10.1038/s41598-024-53149-2

**Published:** 2024-07-04

**Authors:** Mariam Amouzoune, Sajid Rehman, Rachid Benkirane, Sripada Udupa, Sujan Mamidi, Zakaria Kehel, Muamer Al-Jaboobi, Ahmed Amri

**Affiliations:** 1 Faculty of Sciences, University Ibn Tofail, 14000 Kenitra, Morocco; 2 Biodiversity and Crop Improvement Program, International Center for Agricultural Research in the Dry Areas (ICARDA), 10100 Rabat, Morocco; 3 Field Crop Development Center, The Olds College, Lacombe, AB T4L 1W8 Canada; 4 Hudson Alpha Institute for Biotechnology, 601 Genome Way Northwest, Huntsville, AL 35806 USA

**Keywords:** Biotechnology, Genetics, Plant sciences

## Abstract

Leaf rust (LR) caused by *Puccinia hordei* is a serious disease of barley worldwide, causing significant yield losses and reduced grain quality. Discovery and incorporation of new sources of resistance from gene bank accessions into barley breeding programs is essential for the development of leaf rust resistant varieties. To identify Quantitative Trait Loci (QTL) conferring LR resistance in the two barley subsets, the Generation Challenge Program (GCP) reference set of 142 accessions and the leaf rust subset constructed using the Focused Identification of Germplasm Strategy (FIGS) of 76 barley accessions, were genotyped to conduct a genome-wide association study (GWAS). The results revealed a total of 59 QTL in the 218 accessions phenotyped against barley leaf rust at the seedling stage using two *P. hordei* isolates (ISO-SAT and ISO-MRC), and at the adult plant stage in four environments in Morocco. Out of these 59 QTL, 10 QTL were associated with the seedling resistance (SR) and 49 QTL were associated with the adult plant resistance (APR). Four QTL showed stable effects in at least two environments for APR, whereas two common QTL associated with SR and APR were detected on chromosomes 2H and 7H. Furthermore, 39 QTL identified in this study were potentially novel. Interestingly, the sequences of 27 SNP markers encoded the candidate genes (CGs) with predicted protein functions in plant disease resistance. These results will provide new perspectives on the diversity of leaf rust resistance loci for fine mapping, isolation of resistance genes, and for marker-assisted selection for the LR resistance in barley breeding programs worldwide.

## Introduction

Barley (*Hordeum vulgare* ssp. *vulgare* L.) is an important cereal crop worldwide ranking 4th in terms of acreage and providing multiple uses as feed, food, and beverages with its ability to adapt to different harsh climates^1^. In 2018, approximately 2 million ha of barley were harvested in Morocco with an average grain yield of 1.82 t/ha^2^, which is significantly lower compared to the global average grain yield of 2.95 t/ha^2^.This reduced yield may be attributed to several abiotic stresses such as heat drought, as well as biotic stresses, especially foliar diseases. Of these, barley leaf rust, caused by the fungus *Puccinia hordei* Otth, is the most common and widespread foliar disease affecting barley production worldwide. This disease can cause severe yield losses of up to 62% in susceptible varieties under epidemic conditions^3,4^. Genetic resistance to *P. hordei* through the cultivation of resistant varieties is the most economical and environmentally friendly strategy for controlling this disease.

Genetic resistance to leaf rust is generally classified into two forms: seedling resistance (SR) and adult plant resistance (APR). SR is always race-specific, controlled by a single gene with a large effect, and is characterized by a hypersensitive response^5,6^. These genes are easily recognized by breeders and incorporated into the adapted germplasm^7^. In contrast, APR is either specific or non-specific to the pathotypes. Non-specific APR, also known as partial resistance, is often conferred by multiple genes with small effect and is characterized by slow rusting which reduces the severity of the disease^8^. APR is considered more durable than seedling resistance because it remains effective over large areas for long periods of time^9,10^.

Most of the leaf rust resistance genes, also known as *Rph* (resistance to *P. hordei*), are race-specific, conferred by major genes at the seedling stage. To date 28 *Rph* genes conferring LR resistance have been catalogued and mapped^11^, of which 25 (*Rph1*–*Rph19*, *Rph21*, *Rph22*, *Rph25*, *Rph26*, *Rph27*, and *Rph28*) confer seedling resistance^11–15^, and three slow rusting resistance genes namely *Rph20*, *Rph23*, and *Rph24* govern APR^16–18^. Since all SR genes conferring resistance to *P. hordei* are race-specific, these resistance genes lose their effectiveness with the development of new pathotypes/races of the pathogen due to imposed selection pressure by the particular resistance gene. The ineffectiveness of most SR genes and the limited diversity of APR against *P. hordei* highlight the need to diversify genetic resources for resistance to leaf rust in barley by identifying, characterizing, and mapping novel sources of resistance. Therefore, the use of diverse germplasm will be a valuable source to exploit genetic variability for LR resistance and its use in barley breeding programs^19^.

The mining and use of genetic resources is an important element of any crop improvement program. Genebanks provide plant breeders around the world with access to germplasm with valuable traits, such as drought tolerance and resistance to pests and diseases. ICARDA’s genebank contains one of the largest barley collections in the world with more than 32,000 accessions^20^. The approaches for mining plant genetic resources typically use the concept of the core collection, which represents the maximum geographic or morphological diversity as a small subset of 3000 barley accessions which represent 5–10% of the total collection^21,22^. The Generation Challenge Program (GCP) (https://www.generationcp.org) has developed a reference subset of 10% (300 accessions) of the core collection, providing a set of representative germplasm genetic diversity using molecular markers (https://www.genebanks.org/resources/publications/barley-strategy-2008/; accessed on 12 January 2022). Focused Identification of Germplasm Strategy (FIGS) is an alternative approach for efficient exploitation of genetic resources to better respond to seed requests for specific traits sought by breeders. FIGS subsets have been developed for wheat, barley and lentil for various abiotic and biotic stresses. This approach has shown effectiveness in identifying sources of resistance to various diseases and pests such as leaf rust^23^, leaf scald^24^, and net blotch (*Pyrenophora teres Drechs*.)^25^ in barley, powdery mildew^26^, stem rust^27,28^, and yellow rust^29^ in wheat, and *Ascochyta* blight^30^ in lentil.

The deployment of sources of resistance will require the identification and the characterization of QTL. Bi-parental mapping typically focuses on major genes with large effects. It is a time-consuming approach that involves phenotyping successive generations, and it is limited by low allelic diversity and low recombination events, which reduces the mapping resolution. Compared to bi-parental mapping, GWAS is a powerful tool that analyzes the entire genome of a pre-existing set of genetically diverse germplasm to identify SNPs (single nucleotide polymorphism) that are associated with the trait of interest, allowing for a more comprehensive understanding of the genetic architecture of resistance^31^. Several studies have successfully used GWAS to identify genomic regions associated with resistance to several barley diseases including net form of net blotch^32,33^, spot form of net blotch^34,35^, spot blotch^36,37^, leaf rust^38–40^, stem rust^41^, and stripe rust^37,42^. The main objectives of this study were to: (1) identify the genetic loci associated with LR resistance at the seedling and adult plant stages along with their associated candidate genes, and (2) to compare the genetic diversity of FIGS-LR with the GCP subsets and their genomic regions associated with LR resistance, and (3) to identify putative novel genomic loci and confirm known loci conferring leaf rust resistance in barley.

## Results

### Genetic diversity

A total of 218 barley accessions were genotyped using the Illumina iSelect 50K SNP array for barley (Illumina, San Diego, CA, USA) After quality control of removing monomorphic markers, SNPs with the minor allele frequency (MAF) of > 5%, missing data of < 20%, and markers with missing physical map position, 32,686 SNP markers were retained. The chromosomal distribution of the filtered markers used for analysis is shown in Supplementary Fig. S1 in a 1 Mb window size. The SNPs used in this study showed a wide distribution across all the seven barley chromosomes (Table 1). The highest numbers of SNPs (6137) were found on the chromosome 5H, followed by the chromosome 2H (5552), and the chromosome 1H harbored the lowest number of polymorphic markers with 3584 SNPs (Table 1). The minor allele frequency (MAF) ranged from 0.257 to 0.270, with chromosome 6H having the lowest average MAF. Similar PIC values were observed on all chromosomes and an average SNP density of 7.103 SNPs per Mb was calculated (Table 1). The highest number of SNP markers (36.87%) had PIC values greater than 0.3. Gene diversity (GD) ranged from 0.092 to 0.5, with an average of 0.354 (Table 1).

**Table 1:** SNP markers summary statistics and their genome wide distribution of 32,686 filtered SNPs of the 218 barley genotypes.

Chr	No. of SNP markers	Length (Mb)	Density (SNP/Mb)	Gene diversity	PIC	MAF
				Mean	Mean	Mean
1H	3584	558.37	6.42	0.358	0.286	0.268
2H	5552	767.93	7.23	0.352	0.282	0.261
3H	5159	697.85	7.39	0.360	0.288	0.269
4H	3795	646.18	5.87	0.361	0.289	0.270
5H	6137	669.37	9.17	0.351	0.282	0.260
6H	3943	583.02	6.76	0.349	0.281	0.257
7H	4516	656.90	6.87	0.351	0.282	0.259
Genome	32,686	654.233	7.103	0.354	0.284	0.263

The average r^2^ values for SNP markers showed a fast decline with increased physical distance, and the LD decay distance was 250 Kb (r^2^ = 0.2) (Supplementary Fig. S2).

### Population structure

**Figure 1:**
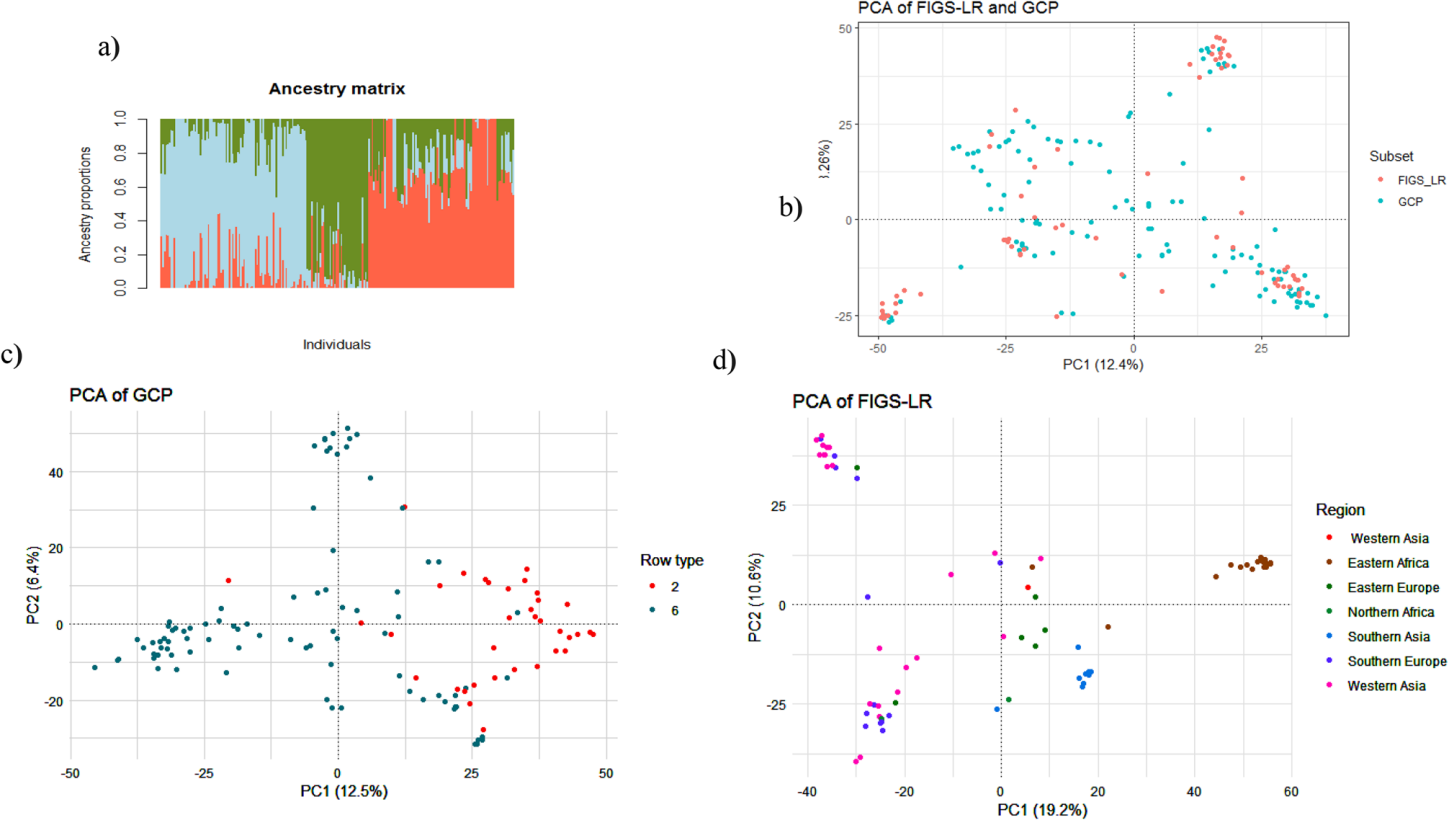
Population structure of FIGS-LR and GCP subsets based on 32,686 SNP markers. Bar plots of individual ancestry proportions for the genetic clusters inferred using LEA package in R (K = 3) for 218 genotypes of FIGS-LR and GCP using 32,686 SNP markers (**a**), scatter plot of the first two principal components (PC1 and PC2) of the 218 barley accessions clustered based on the subset (**b**), GCP subset based on row type (**c**), and FIGS-LR based on the geographic origin (**d**).

The genotyping data of 32,686 filtered SNPs was used to analyze the population structure of the 218 barley genotypes of FIGS-LR and GCP. Figure 1a shows the admixture bar plots of four clusters represented by four different colors. In addition, the principal component analysis (PCA) was performed to explore patterns of genetic variation within and between subsets using the genotypic data. Based on the subset (FIGS_LR and GCP) information of each genotype, the PCA scatter plot showed that the first two principal components PC1 and PC2 accounted for 12.4% and 8.26% respectively (Fig. 1b). The analyzed barley accessions did not cluster exclusively by subset (FIGS_LR and GCP); the first cluster contained mainly GCP accessions, but also some FIGS_LR accessions. The second cluster contained predominantly accessions from FIGS_LR, which are mainly distributed in the extremities.

The genetic variation within subsets was analyzed using PCA. The results showed that the GCP subset exhibited a clustering pattern based on the row type, with little admixture observed with PC1 and PC2, which explained 12.5% and 6.4% of the total variation, respectively (Fig. 1c). In contrast, FIGS-LR subset did not show any clustering pattern based on the row type, but it exhibited a clustering pattern based on geographical origin, with clear clusters observed for the accessions from Eastern Africa and Southern Asia. The first two principal components of FIGS-LR explained a higher proportion of the total variation (30.8%) compared to the GCP subset (18.9%), with PC1 and PC2 explaining 19.2% and 10.6% of the total variation, respectively (Fig. 1d).

### GWAS of leaf rust resistance

Genome-wide association study (GWAS) for resistance to barley leaf rust at the seedling and at the adult plant stages was conducted using a total of 218 barley accessions and 32,686 SNP markers. The QQ plots for LR resistance are shown in Supplementary Fig. S3. The QQ plots were used to check the fitness of the six models implemented in GAPIT3 across all environments and isolates, and the MLM model accounting for population structure and relatedness (PCA + K) was the best fit model for leaf rust resistance for both *Ph* isolates at SR and for APR in all environments (Supplementary Fig. S4). This study identified 10 and 49 QTL (− log10 (p values) ≥ 3.3) associated with *P. hordei* resistance at the seedling and at the adult plant stages, respectively (Tables 2, 3). For LR resistance at the seedling stage, 4 QTL associated with ISO-SAT isolate were detected on chromosomes 3H, and 6H (Table 2), with JHI-Hv50k-2016-189805 being the most significant SNP marker with the lowest p-value of 2.06 × 10^–04^, the highest effect of − 0.266, and the highest R^2^ of 5.43% on chromosome 3H. For the ISO-MRC isolate, 6 QTL were detected on chromosomes 1H, 2H, and 7H. The QTL *QPh.ISO-MRC-5* on 2H (644,195,113 bp) was the most significant QTL with the lowest p-value of 8.852 × 10^–05^ and the highest R^2^ of 7.541%. The highest effect (− 0.387) was caused by the QTL *QPh.ISO-MRC-4* on the chromosome 2H at 623,237,567 bp.

**Table 2:** Summary of genomic regions significantly associated with the seedling resistance to *Puccinia hordei* in FIGS-LR and GCP barley subsets.

QTL	Marker	Chr^a^	Position^b^	p.value	FDR	R2 (%)	Effect^c^	Allele frequency of GCP	Allele frequency of FIGS
SR-MRC									
*QPh.ISO-MRC-1*	JHI-Hv50k-2016-5493	1	5,345,263	3.06E−04	1.224E−05	6.36	G(− 0.201)	19.72	26.32
*QPh.ISO-MRC-2*	JHI-Hv50k-2016-108241	2	573,521,745	5.47E−04	1.989E−05	5.81	T(0.205)	33.1	38.16
*QPh.ISO-MRC-3*	JHI-Hv50k-2016-108614	2	574,568,303	5.28E−04	1.53E−05	5.85	G(0.218)	59.86	60.53
*QPh.ISO-MRC-4*	JHI-Hv50k-2016-124322	2	623,237,567	1.55E−04	6.119E−06	7.01	A(− 0.388)	90.14	96.05
*QPh.ISO-MRC-5*	JHI-Hv50k-2016-135641**	2	644,195,113	8.85E−05	1.53E−06	7.54	A(− 0.251)	76.76	76.32
*QPh.ISO-MRC-6*	JHI-Hv50k-2016-491720**	7	549,976,599	2.88E−04	7.649E−06	6.42	G(0.316)	88.03	97.37
SR-SAT									
*QPh.ISO-SAT-1*	JHI-Hv50k-2016-189805	3	487,537,283	2.06E−04	3.059E−06	5.43	A(− 0.266)	21.13	23.68
*QPh.ISO-SAT-2*	JHI-Hv50k-2016-373247	6	12,265,667	4.53E−04	9.178E−06	4.83	A(− 0.214)	58.45	47.37
*QPh.ISO-SAT-3*	JHI-Hv50k-2016-404658	6	407,625,773	4.50E−04	7.649E−06	4.84	G(0.211)	31.69	35.53
*QPh.ISO-SAT-4*	JHI-Hv50k-2016-433188	6	560,082,939	3.40E−04	6.119E−06	5.05	C(− 0.194)	60.56	51.32

**Common QTl between SR and APR.

^a^Chromosome.

^b^Physical position of SNPs based on Morex genome version 3.0

^c^Allele effect. The p-value threshold (− log10 p-value (0.0005) ≥ 3.3) was used to declare significant QTL.

**Table 3:** Summary of genomic regions significantly associated with the adult plant resistance to *Puccinia hordei* in FIGS-LR and GCP barley subsets.

QTL	Marker	Chr^a^	Position^b^	p.value	FDR	R2 (%)	Effect^c^	Allele frequency of GCP (%)	Allele frequency of FIGS (%)
SAT2017									
*QPh.SAT2017-1*	JHI-Hv50k-2016-229639	4	10,008,919	1.13E−04	6.12E−06	6.71	A(− 14.406)	98.59	86.84
*QPh.SAT2017-2*	JHI-Hv50k-2016-335902*	5	528,058,903	4.59E−04	1.53E−05	5.49	G(8.078)	84.51	61.84
*QPh.SAT2017-3*	JHI-Hv50k-2016-461706	7	50,031,910	4.22E−04	1.38E−05	5.56	C(− 8.005)	86.62	77.63
*QPh.SAT2017-4*	JHI-Hv50k-2016-511346	7	613,938,104	2.94E−04	1.07E−05	5.88	T(9.512)	87.32	64.47
SAT2018									
*QPh.SAT2018-1*	JHI-Hv50k-2016-41535	1	475,085,011	4.71E−04	8.57E−05	3.99	C(− 11.422)	9.15	25
	JHI-Hv50k-2016-41672	1	475,371,901	8.96E−05	3.21E−05	5.05	G(10.907)	8.45	25
*QPh.SAT2018-2*	JHI-Hv50k-2016-86045	2	93,370,745	1.07E−05	1.38E−05	6.45	A(− 9.977)	7.04	7.89
*QPh.SAT2018-3*	JHI-Hv50k-2016-135076**	2	643,275,772	3.64E−04	6.73E−05	4.15	T(13.281)	4.23	6.58
*QPh.SAT2018-4*	JHI-Hv50k-2016-138543	2	649,502,617	3.90E−06	6.12E−06	7.13	C(10.079)	93.66	89.47
*QPh.SAT2018-5*	BOPA2_12_11295	3	186,046,917	4.41E−04	7.95E−05	4.03	G(7.516)	10.56	9.21
*QPh.SAT2018-6*	JHI-Hv50k-2016-205616	3	564,696,164	4.14E−04	7.34E−05	4.07	T(6.837)	28.87	60.53
*QPh.SAT2018-7*	SCRI_RS_65010	3	598,142,453	3.93E−04	7.19E−05	4.1	A(− 7.516)	19.01	27.63
*QPh.SAT2018-8*	JHI-Hv50k-2016-246896	4	459,723,972	3.10E−05	2.14E−05	5.74	T(8.321)	18.31	17.11
*QPh.SAT2018-9*	JHI-Hv50k-2016-247049	4	461,112,374	2.83E−04	5.97E−05	4.31	G(7.311)	18.31	17.11
*QPh.SAT2018-10*	JHI-Hv50k-2016-247160	4	461,874,702	2.83E−04	5.51E−05	4.31	A(− 7.311)	18.31	17.11
*QPh.SAT2018-11*	JHI-Hv50k-2016-247277	4	463,152,295	2.83E−04	5.66E−05	4.31	A(− 7.311)	18.31	17.11
*QPh.SAT2018-12*	JHI-Hv50k-2016-265580	4	587,293,277	5.12E−04	9.33E−05	3.94	G(− 16.566)	95.77	77.63
*QPh.SAT2018-13*	SCRI_RS_141803	4	589,422,387	4.87E−04	9.03E−05	3.97	A(− 6.465)	70.42	81.58
*QPh.SAT2018-14*	JHI-Hv50k-2016-271709	4	600,927,835	9.43E−05	3.37E−05	5.02	A(− 6.057)	25.35	28.95
	JHI-Hv50k-2016-272051	4	601,538,674	3.02E−07	3.06E−06	8.91	C(− 9.73)	83.1	84.21
	JHI-Hv50k-2016-272121	4	601,760,759	2.46E−06	4.59E−06	7.45	G(9.13)	80.99	82.89
	SCRI_RS_188829	4	602,053,544	2.76E−05	1.99E−05	5.82	A(− 8.064)	83.8	84.21
*QPh.SAT2018-15*	JHI-Hv50k-2016-304171	5	394,402,295	5.23E−05	2.91E−05	5.4	G(− 7.674)	71.13	78.95
	SCRI_RS_236759	5	394,808,984	2.20E−05	1.53E−05	5.97	A(− 8.026)	71.13	77.63
*QPh.SAT2018-16*	BOPA2_12_11298*	5	514,627,876	5.33E−04	1.04E−04	3.91	A(− 14.212)	8.45	31.58
*QPh.SAT2018-17*	JHI-Hv50k-2016-337389	5	530,890,859	4.22E−04	7.50E−05	4.06	T(10.03)	21.83	28.95
*QPh.SAT2018-18*	JHI-Hv50k-2016-339430	5	536,277,945	6.74E−05	3.06E−05	5.23	G(− 9.013)	8.45	14.47
*QPh.SAT2018-19*	JHI-Hv50k-2016-344094	5	545,187,485	1.75E−04	4.59E−05	4.62	A(− 9.127)	9.15	18.42
*QPh.SAT2018-20*	JHI-Hv50k-2016-344917	5	546,907,629	4.42E−04	8.11E−05	4.03	C(− 6.01)	23.24	19.74
SAT2019									
*QPh.SAT2019-1*	JHI-Hv50k-2016-37820*	1	458,868,886	1.86E−04	1.53E−05	9.77	G(6.975)	7.04	7.89
*QPh.SAT2019-2*	JHI-Hv50k-2016-46373	1	486,477,700	3.26E−04	3.06E−05	8.99	A(− 8.298)	9.15	0
	JHI-Hv50k-2016-46552	1	486,668,358	3.26E−04	2.91E−05	8.99	T(8.298)	9.86	2.63
*QPh.SAT2019-3*	JHI-Hv50k-2016-57599	1	515,112,071	3.93E−04	3.37E−05	8.74	T(4.519)	34.51	22.37
*QPh.SAT2019-4*	SCRI_RS_135391	2	11,532,558	5.18E−04	3.98E−05	8.37	A(− 5.842)	21.83	34.21
*QPh.SAT2019-5*	JHI-Hv50k-2016-110935	2	593,971,328	9.79E−05	1.53E−06	10.66	A(− 5.101)	50	76.32
	JHI-Hv50k-2016-111047	2	593,922,554	9.79E−05	7.65E−06	10.66	T(5.101)	51.41	76.32
*QPh.SAT2019-6*	JHI-Hv50k-2016-116579	2	608,480,345	1.20E−04	1.07E−05	10.37	A(− 5.457)	26.06	30.26
*QPh.SAT2019-7*	JHI-Hv50k-2016-127870	2	630,365,638	5.47E−04	4.28E−05	8.29	C(− 9.945)	11.97	10.53
	JHI-Hv50k-2016-127929	2	630,599,862	5.47E−04	4.44E−05	8.29	C(− 9.945)	11.97	6.58
*QPh.SAT2019-8*	JHI-Hv50k-2016-207495	3	574,021,612	1.66E−04	1.38E−05	9.92	C(− 8.519)	11.27	3.95
*QPh.SAT2019-9*	JHI-Hv50k-2016-226348*	4	200,043	4.19E−04	3.82E−05	8.65	C(− 5.066)	76.76	72.37
	JHI-Hv50k-2016-226367*	4	210,117	1.97E−04	1.84E−05	9.68	T(5.284)	74.65	71.05
*QPh.SAT2019-10*	JHI-Hv50k-2016-329065*	5	516,956,372	2.76E−04	2.75E−05	9.22	A(− 8.887)	7.04	1.32
*QPh.SAT2019-11*	BOPA2_12_30596	6	184,345,417	2.04E−04	2.29E−05	9.63	A(− 10.089)	7.04	1.32
*QPh.SAT2019-12*	JHI-Hv50k-2016-395409	6	270,437,155	2.04E−04	2.45E−05	9.63	C(− 10.089)	7.04	1.32
*QPh.SAT2019-13*	JHI-Hv50k-2016-395444	6	273,286,262	2.04E−04	2.14E−05	9.63	G(10.089)	7.04	1.32
*QPh.SAT2019-14*	JHI-Hv50k-2016-502834	7	596,652,222	4.11E−04	3.52E−05	8.68	C(− 5.574)	13.38	15.79
GUICH2018									
*QPh.GUICH2018-1*	JHI-Hv50k-2016-38800*	1	464,120,530	8.22E−05	1.22E−05	7.42	T(3.653)	35.92	32.89
*QPh.GUICH2018-2*	JHI-Hv50k-2016-69628	2	17,690,886	1.80E−04	2.14E−05	6.69	C(4.698)	13.38	6.58
*QPh.GUICH2018-3*	BOPA2_12_20326	2	46,947,830	4.78E−04	4.13E−05	5.79	G(5.502)	92.25	84.21
*QPh.GUICH2018-4*	JHI-Hv50k-2016-131955	2	637,682,135	5.94E−05	6.12E−06	7.72	C(− 5.385)	5.63	6.58
*QPh.GUICH2018-5*	JHI-Hv50k-2016-141021	2	653,624,675	1.91E−04	2.45E−05	6.63	A(− 3.981)	16.2	6.58
*QPh.GUICH2018-6*	JHI-Hv50k-2016-185883	3	460,861,131	5.09E−04	4.28E−05	5.73	C(− 4.931)	9.15	3.95
*QPh.GUICH2018-7*	JHI-Hv50k-2016-228182*	4	5,777,486	9.57E−05	1.84E−05	7.28	T(5.572)	8.45	3.95
*QPh.GUICH2018-8*	JHI-Hv50k-2016-333100	5	523,740,008	9.45E−07	1.53E−06	11.75	C(− 4.261)	42.25	30.26
*QPh.GUICH2018-9*	JHI-Hv50k-2016-333747*	5	524,805,000	4.72E−04	3.82E−05	5.8	C(− 3.933)	66.9	57.89
*QPh.GUICH2018-10*	JHI-Hv50k-2016-384829	6	41,559,241	4.75E−04	3.98E−05	5.79	T(3.603)	16.2	17.11
*QPh.GUICH2018-11*	JHI-Hv50k-2016-491548**	7	547,518,640	8.99E−05	1.38E−05	7.33	T(5.172)	11.97	5.26
	JHI-Hv50k-2016-491670**	7	548,075,313	2.41E−05	3.06E−06	8.58	C(− 5.422)	11.97	6.58

*Common QTL in at least two environments.

**Common genomic regions between SR and APR.

^a^Chromosome.

^b^Physical position of SNPs based on Morex genome version 3.

^c^Allele effect. The p-value threshold (−log10 p-value (0.0005) ≥ 3.3) was used to declare significant QTL.

For LR resistance at the adult plant stage, 49 QTL were identified using phenotypic data of four field environments. Among them, 4, 22, and 14 QTL were detected in Sidi Allal Tazi station in 2017 (SAT2017), 2018 (SAT2018), and in 2019 (SAT2019) respectively. Additionally, 11 QTL were identified in GUICH2018 (Table 3). The total phenotypic variance explained by the SNP markers associated with APR was 0.236, 1.260, 1.682 and 0.865 for SAT2017, SAT2018, GUICH2018, and SAT2019, respectively.

Among the 49 QTL associated with APR, four QTL were stable in at least two environments. These QTL were namely *QPh.SAT2019-1* and *QPh.GUICH2018-1* detected on the chromosome 1H at 458,868,886 bp and 464,120,530 bp respectively, *QPh.SAT2019-9* (200,043–210,117 bp) and *QPh.GUICH2018-7* (5,777,486 bp) on 4H, the two QTL *QPh.SAT2018-16* (514,627,876 bp) and *QPh.SAT2019-10* (516,956,372 bp), and *QPh.GUICH2018-9* (524,805,000 bp), *QPh.SAT2017-2* (528,058,903 bp) detected on 5H (Table 3).

Two common QTL associated with LR were detected for both SR and APR. The first one associated with the SR QTL *QPh.ISO-MRC-5* at 644,195,113 bp and the APR QTL *QPh.SAT2018-3* at 643,275,772 bp on chromosome 2H, the second one on chromosome 7H associated with the the SR QTL *QPh.ISO-MRC-6* at 549,976,599 bp and APR QTL *QPh.GUICH2018-11* (547,518,640–548,075,313 bp).

### QTL alignments and candidate genes (CGs)

Several studies have identified *Ph* resistance loci, which allows for a comparison of these loci to those identified in the present study. Interestingly, out of the 59 QTL identified, 21 overlapped with the known genes or QTL involved in LR resistance (Table 4), while 39 QTL were located at chromosomal positions that had not previously been reported to be involved in *Ph* resistance. Therefore, these 39 QTL could be considered novel loci for *Ph* resistance.

**Table 4:** QTL alignment and candidate genes identified for seedling and adult plant resistance to leaf rust in both barley subsets FIGS-LR and GCP.

QTL	Marker	Chr^a^	Position^b^	Candidate genes	Homology	Previously identified gene/QTL^c^
Seedling resistance						
ISO-MRC						
*QPh.ISO-MRC-1*	JHI-Hv50k-2016–5493	1	5,345,263	HORVU.MOREX.r3.1HG0002590	Serine/threonine-protein kinase 19	*Rph4.d; Rphq14*; JHI-Hv50k-2016-5369^43–45^
*QPh.ISO-MRC-2*	JHI-Hv50k-2016-108241	2	573,521,745	HORVU2Hr1G092880.7	–	–
*QPh.ISO-MRC-3*	JHI-Hv50k-2016-108614	2	574,568,303	HORVU.MOREX.r3.2HG0185460	Alpha/beta-Hydrolases superfamily protein, putative	–
*QPh.ISO-MRC-4*	JHI-Hv50k-2016-124322	2	623,237,567	HORVU.MOREX.r3.2HG0200040	Phospholipid/glycerol acyltransferase family protein	–
*QPh.ISO-MRC-5*	JHI-Hv50k-2016-135641**	2	644,195,113	HORVU.MOREX.r3.2HG0208800	Actin-related family protein	–
*QPh.ISO-MRC-6*	JHI-Hv50k-2016-491720**	7	549,976,599	HORVU.MOREX.r3.7HG0724110	RING/U-box superfamily protein	–
ISO-SAT						
*QPh.ISO-SAT-1*	JHI-Hv50k-2016-189805	3	487,537,283	HORVU.MOREX.r3.3HG0289030	Neuronal acetylcholine receptor subunit alpha-5	JHI-Hv50k-2016-189928^45^
*QPh.ISO-SAT-2*	JHI-Hv50k-2016-373247	6	12,265,667	MLOC_1411	–	ABC152A; *Rphq15*^45^
*QPh.ISO-SAT-3*	JHI-Hv50k-2016-404658	6	407,625,773	HORVU.MOREX.r3.6HG0600090	Kinase family protein	–
*QPh.ISO-SAT-4*	JHI-Hv50k-2016-433188	6	560,082,939	HORVU.MOREX.r3.6HG0633530	ATP-dependent RNA helicase	JHI-Hv50k-2016-431728^45^
Adult plant resistance						
SAT2017						
*QPh.SAT2017-1*	JHI-Hv50k-2016-229639	4	10,008,919	HORVU.MOREX.r3.4HG0334930	Inositol hexakisphosphate and diphosphoinositol-pentakisphosphate kinase	–
*QPh.SAT2017-2*	JHI-Hv50k-2016-335902*	5	528,058,903	HORVU.MOREX.r3.5HG0511140	Cysteine protease	Rph9.i^44^
*QPh.SAT2017-3*	JHI-Hv50k-2016-461706	7	50,031,910	HORVU7Hr1G027540.1	Glutathione S-transferase family protein	–
*QPh.SAT2017-4*	JHI-Hv50k-2016-511346	7	613,938,104	HORVU.MOREX.r3.7HG0744530	Disease resistance protein (TIR-NBS-LRR class)	*RphQ14* (*Rph3/19*); *Rph3.c*; *RphQ28*; *Rph3.c*; JHI-Hv50k-2016-511500; JHI-Hv50k-2016-510780^44–47^
SAT2018						
*QPh.SAT2018-1*	JHI-Hv50k-2016-41535	1	475,085,011	HORVU.MOREX.r3.1HG0076530	ATP-dependent 6-phosphofructokinase	JHI-Hv50k-2016-41795^45^
	JHI-Hv50k-2016-41672	1	475,371,901	HORVU.MOREX.r3.1HG0076580	ATP-binding cassette transporter subfamily A member	JHI-Hv50k-2016-41795^45^
*QPh.SAT2018-2*	JHI-Hv50k-2016-86045	2	93,370,745	HORVU.MOREX.r3.2HG0124130	Cyclin family protein	–
*QPh.SAT2018-3*	JHI-Hv50k-2016-135076**	2	643,275,772	HORVU.MOREX.r3.2HG0208330	Serine/threonine-protein kinase	–
*QPh.SAT2018-4*	JHI-Hv50k-2016-138543	2	649,502,617	MLOC_36886	Response regulator 2	*QPh.2H-2*; *QRph.sun-2H.2*^48^
*QPh.SAT2018-5*	BOPA2_12_11295	3	186,046,917	HORVU.MOREX.r3.3HG0255150	Sec14p-like phosphatidylinositol transfer family protein	–
*QPh.SAT2018-6*	JHI-Hv50k-2016-205616	3	564,696,164	HORVU.MOREX.r3.3HG0307410	DNA ligase-like	Bmag606^49^
*QPh.SAT2018-7*	SCRI_RS_65010	3	598,142,453	HORVU.MOREX.r3.3HG0320950	Villin	–
*QPh.SAT2018-8*	JHI-Hv50k-2016-246896	4	459,723,972	HORVU.MOREX.r3.4HG0385950	Elongator complex protein 6	–
*QPh.SAT2018-9*	JHI-Hv50k-2016-247049	4	461,112,374	HORVU.MOREX.r3.4HG0386090	Glutathione-S-transferase	–
*QPh.SAT2018-10*	JHI-Hv50k-2016-247160	4	461,874,702	HORVU.MOREX.r3.4HG0386280	Ribosomal protein S24e family protein	–
*QPh.SAT2018-11*	JHI-Hv50k-2016-247277	4	463,152,295	HORVU.MOREX.r3.4HG0386480	Calcium-transporting ATPase	–
*QPh.SAT2018-12*	JHI-Hv50k-2016-265580	4	587,293,277	HORVU.MOREX.r3.4HG0409850	4-coumarate–CoA ligase-like 4	–
*QPh.SAT2018-13*	SCRI_RS_141803	4	589,422,387	HORVU.MOREX.r3.4HG0410720	Alpha-2C adrenergic receptor	–
*QPh.SAT2018-14*	JHI-Hv50k-2016-271709	4	600,927,835	HORVU.MOREX.r3.4HG0415080	Mediator of RNA polymerase II transcription subunit 12	–
	JHI-Hv50k-2016-272051	4	601,538,674	HORVU.MOREX.r3.4HG0415230	basic helix-loop-helix (bHLH) DNA-binding superfamily protein	–
	JHI-Hv50k-2016-272121	4	601,760,759	HORVU.MOREX.r3.4HG0415340	ATP-binding cassette transporter subfamily A member	–
	SCRI_RS_188829	4	602,053,544	HORVU.MOREX.r3.4HG0415420	Tubulin beta chain	–
*QPh.SAT2018-15*	JHI-Hv50k-2016-304171	5	394,402,295	HORVU.MOREX.r3.5HG0474140	Cytochrome B5-like protein	*Rph2.t*^44^
	SCRI_RS_236759	5	394,808,984	HORVU.MOREX.r3.5HG0474200	BTB/POZ domain containing protein, expressed	*Rph2.t*^44^
*QPh.SAT2018-16*	BOPA2_12_11298*	5	514,627,876	HORVU.MOREX.r3.5HG0505140	Phosphatase 2C family protein	–
*QPh.SAT2018-17*	JHI-Hv50k-2016-337389	5	530,890,859	HORVU.MOREX.r3.5HG0512320	Cytochrome P450	JHI-Hv50k-2016-338772; *RphQ25; QLr.S42-5H.a; Rph9; Rph12*^45,47,50,51^
*QPh.SAT2018-18*	JHI-Hv50k-2016-339430	5	536,277,945	HORVU.MOREX.r3.5HG0514020	GRAS transcription factor	JHI-Hv50k-2016-338772; *RphQ25; QLr.S42-5H.a; Rph9; Rph12*^45,47,50,51^
*QPh.SAT2018-19*	JHI-Hv50k-2016-344094	5	545,187,485	HORVU.MOREX.r3.5HG0518020	Lipid transfer protein	JHI-Hv50k-2016-338772; *RphQ25; QLr.S42-5H.a; Rph9; Rph12*^45,47,50,51^
*QPh.SAT2018-20*	JHI-Hv50k-2016-344917	5	546,907,629	HORVU.MOREX.r3.5HG0518820	Translation elongation factor EF1B, gamma chain	–
SAT2019						
*QPh.SAT2019-1*	JHI-Hv50k-2016-37820*	1	458,868,886	HORVU.MOREX.r3.1HG0072590	Aminotransferase-related family protein	–
*QPh.SAT2019-2*	JHI-Hv50k-2016-46373	1	486,477,700	HORVU.MOREX.r3.1HG0080620	Exostosin-2	–
	JHI-Hv50k-2016-46552	1	486,668,358	HORVU.MOREX.r3.1HG0080690	Endoglucanase	–
*QPh.SAT2019-3*	JHI-Hv50k-2016-57599	1	515,112,071	HORVU.MOREX.r3.1HG0094980	Mitochondrial outer membrane porin	11_20509^52^
*QPh.SAT2019-4*	SCRI_RS_135391	2	11,532,558	HORVU.MOREX.r3.2HG0100410	tRNA/tmRNA (uracil-C(5))-methyltransferase	–
*QPh.SAT2019-5*	JHI-Hv50k-2016-110935	2	593,971,328	HORVU.MOREX.r3.2HG0188960	BEACH domain-containing protein	JHI-Hv50k-2016-111819; JHI-Hv50k-2016-110777; JHI-Hv50k-2016-111042; *Rphq11*^43,45^
	JHI-Hv50k-2016-111047	2	593,922,554	HORVU.MOREX.r3.2HG0188940	Gag polyprotein	JHI-Hv50k-2016-111819; JHI-Hv50k-2016-110777; JHI-Hv50k-2016-111042; *Rphq11*^43,45^
*QPh.SAT2019-6*	JHI-Hv50k-2016-116579	2	608,480,345	HORVU.MOREX.r3.2HG0194000	p-loop containing nucleoside triphosphate hydrolases superfamily protein, putative	–
*QPh.SAT2019-7*	JHI-Hv50k-2016-127870	2	630,365,638	HORVU.MOREX.r3.2HG0202810	Receptor-like kinase	–
	JHI-Hv50k-2016-127929	2	630,599,862	HORVU.MOREX.r3.2HG0202930	Transmembrane emp24 domain-containing protein	–
*QPh.SAT2019-8*	JHI-Hv50k-2016-207495	3	574,021,612	HORVU.MOREX.r3.3HG0310540	Mannosyl-oligosaccharide glucosidase	–
*QPh.SAT2019-9*	JHI-Hv50k-2016-226348*	4	200,043	HORVU.MOREX.r3.4HG0331240	Cytochrome oxidase complex assembly protein	–
	JHI-Hv50k-2016-226367*	4	210,117	HORVU.MOREX.r3.4HG0331260	ABC transporter ATP-binding protein ARB1	–
*QPh.SAT2019-10*	JHI-Hv50k-2016-329065*	5	516,956,372	HORVU.MOREX.r3.5HG0505960	Zinc finger family protein	*Rph9.i*^44^
*QPh.SAT2019-11*	BOPA2_12_30596	6	184,345,417	HORVU.MOREX.r3.6HG0577760	Ran-binding zinc finger protein	–
*QPh.SAT2019-12*	JHI-Hv50k-2016-395409	6	270,437,155	HORVU.MOREX.r3.6HG0585520	Calmodulin binding protein-like protein	–
*QPh.SAT2019-13*	JHI-Hv50k-2016-395444	6	273,286,262	HORVU.MOREX.r3.6HG0585880	Flavin-containing monooxygenase	bPb-3722^46^
*QPh.SAT2019-14*	JHI-Hv50k-2016-502834	7	596,652,222	HORVU.MOREX.r3.7HG0736860	Aspartyl/glutamyl-tRNA(Asn/Gln) amidotransferase subunit B, putative isoform 2	*QPh.7H-3; Rphq9; QTL_Castro*; JHI-Hv50k-2016-501477^48,49,53^
GUICH2018						
*QPh.GUICH2018-1*	JHI-Hv50k-2016-38800*	1	464,120,530	MLOC_60455	BHLH transcription factor 1	–
*QPh.GUICH2018-2*	JHI-Hv50k-2016-69628	2	17,690,886	HORVU.MOREX.r3.2HG0104340	Integrator complex subunit 11	–
*QPh.GUICH2018-3*	BOPA2_12_20326	2	46,947,830	MLOC_55107	Xylose isomerase	–
*QPh.GUICH2018-4*	JHI-Hv50k-2016-131955	2	637,682,135	HORVU.MOREX.r3.2HG0205670	Root meristem growth factor 9	–
*QPh.GUICH2018-5*	JHI-Hv50k-2016-141021	2	653,624,675	HORVU.MOREX.r3.2HG0213170	Ubiquinol oxidase	–
*QPh.GUICH2018-6*	JHI-Hv50k-2016-185883	3	460,861,131	HORVU.MOREX.r3.3HG0284730	B3 domain transcription factor	–
*QPh.GICH2018-7*	JHI-Hv50k-2016-228182*	4	5,777,486	HORVU.MOREX.r3.4HG0333480	Globulin-1	–
*QPh.GUICH2018-8*	JHI-Hv50k-2016-333100	5	523,740,008	HORVU5Hr1G093890.1	Undescribed protein	–
*QPh.GUICH2018-9*	JHI-Hv50k-2016-333747*	5	524,805,000	HORVU.MOREX.r3.5HG0509760	Auxin-responsive protein	*Rph9.i*^44^
*QPh.GUICH2018-10*	JHI-Hv50k-2016-384829	6	41,559,241	HORVU.MOREX.r3.6HG0554800	Transmembrane protein 53	*QPh.6H-2; Rphq3; QTL_Castro*; *RphQ11; QTL_Hickey; Rph24*; *QRph-6H.49*^16,18,46,49,53,54^
*QPh.GUICH2018-11*	JHI-Hv50k-2016-491548**	7	547,518,640	HORVU.MOREX.r3.7HG0723480	F-box family protein	–
	JHI-Hv50k-2016-491670**	7	548,075,313	HORVU7Hr1G093380	dihydroflavonol 4-reductase-like1	–

*Common QTL in at least two environments.

**Common genomic regions between SR and APR.

^a^Chromosome.

^b^Physical position of SNPs based on Morex genome version 3.

^c^QTL previously mapped at the same position for barley leaf rust. The p-value threshold (− log10 p-value (0.0005) ≥ 3.3) was used to declare significant QTL.

The putative CGs associated with the significant SNP markers for LR resistance at both SR and APR have been reported in Table 4. Most of the CGs were located in genomic regions encoding proteins with functional domains involved in plant defense mechanisms based on their annotation in the barley reference genome. In total, we have reported 25 SNP markers, which showed homologies with functional proteins/enzymes related to disease resistance (Table 4).

## Discussion

Leaf rust is an important disease of barley that can severely reduce barley productivity of susceptible cultivars. The rapid evolution of new virulent races renders most of the resistance genes ineffective. Therefore, a continuous search and identification of novel sources of resistance conserved in different genebanks is required for the diversification of genetic resistance. Both FIGS and GCP barley subsets have proven to be powerful approaches for identifying sources of resistance to *Puccinia hordei* at the seedling and adult plant stages^23^. Hence, it was important to characterize the resistance loci/genes available in germplasm before being exploited by breeding programs. In this study, we explored the genetic diversity of FIGS-LR and GCP subsets by genotyping them with 50 k SNP array followed by association mapping of leaf rust resistance at the seedling and adult plant stages.

The filtered genotyping data (32,686 SNPs) was used to estimate the GD (gene diversity) and PIC and to identify QTL associated with LR resistance at the seedling and at the adult plant stages. The PIC can be used to measure the ability of a marker to detect polymorphisms, thus, the PIC can be divided into three categories: highly polymorphic marker when PIC > 0.5, moderately informative marker when 0.25 < PIC < 0.5, and low-informative marker when PIC < 0.25^55^. In our study, the mean PIC value was 0.284, indicating that most of the SNP markers used in this study were moderately informative markers. This value was similar to those reported in previous studies^56,57^. Similar PIC values in all chromosomes indicate that the SNP markers detected a consistent level of genetic diversity across all seven barley chromosomes. Furthermore, the average gene diversity (GD) value was 0.354. These findings are similar to those reported by Ref.^58^ using SNP markers (GD = 0.35).

Population structure analysis typically grouped barley accessions based on row type (2- or 6-row). PCA does not always classify accessions into distinct groups, especially when accessions are from diverse geographical origins^59^. The genetic variation within the GCP and FIGS-LR subsets analyzed using PCA suggests that the row type has some influence on the genetic variation observed in the GCP, while the geographic origin has some effect on FIGS-LR. The first two PCs of FIGS-LR explained higher proportion of the total variation compared with GCP subset (Fig. 1c,d). The observed patterns of genetic variation in each subset may be attributed to their respective assembling strategies. For example, the co-evolutionary trait-environment approach of FIGS-LR^28,60^ may have resulted in a subset of accessions that are more similar to each other based on the specific trait and environment they were selected for, while the GCP subset, which aims to capture maximum genetic diversity, may have resulted in a more diverse set of accessions with variation influenced by other factors such as geographic origin or environmental adaptation. Similarly, Muñoz-Amatriaín et al.^52^ reported that principal component analysis identified five major subpopulations within a core collection of 2417 barley accessions which was genotyped with 7842 SNPs. It differentiated mainly by geographical origin and row type.

To identify SNP markers associated with LR resistance in the two barley subsets, genome-wide association studies were conducted using phenotyping data of 218 barley accessions and filtered genotyping data (32,686 SNPs). Earlier studies have demonstrated the efficiency of GWAS for genetic mapping of disease resistance to net form net blotch^32^, spot form net blotch^34,35^, spot blotch^36,37^, leaf rust^46^, stem rust^41^, and stripe rust^37,42^. GWAS analysis revealed a total of 59 QTL. Among these, 10 QTL were associated with the SR and 49 QTL were associated with the APR. Four QTL showed stable effects in at least two environments for APR, while two common QTL associated with SR and APR were detected on chromosomes 2H and 7H. Furthermore 39 (66%) QTL identified in this study were potentially novel loci, which is relatively higher compared to the novel QTL identified in AM2017, where 32 (55%) novel QTL were found^45^. The SR GWAS identified 10 QTL associated with LR resistance, among which 4 QTL were aligned to previously reported LR QTL, and 6 QTL were novel. On chromosome 1H, only one QTL (*QPh.ISO-MRC-1*) was associated with the *Ph* isolate ISO-MRC at 5,345,263 bp. This QTL was colocalized with a known *Ph* resistance loci^43–45^. The frequency of the favorable allele at this QTL was higher in FIGS-LR accessions with 26.32% compared to GCP with 19.72%. Four new QTL (*QPh.ISO-MRC-2, QPh.ISO-MRC-3, QPh.ISO-MRC-4 and QPh.ISO-MRC-5*) were found to be significantly associated with SR (ISO-MRC) on chromosome 2H. The favorable allele frequencies of these three QTL *QPh.ISO-MRC-2, QPh.ISO-MRC-3 and QPh.ISO-MRC-4* were slightly higher in FIGS-LR compared to GCP, while for the QTL *QPh.ISO-MRC-5*, the favorable allele was equal for both subsets. The QTL *QPh.ISO-SAT-1* (487,537,283 bp) was the only QTL associated with SR on chromosome 3H. This QTL coincided with a MTA at 488,214,308 bp identified in a previous study^45^. Furthermore, three QTL (*QPh.ISO-SAT-2, QPh.ISO-SAT-3 and QPh.ISO-SAT-4*) were associated with SR against *Ph* isolate ISO-SAT on chromosome 6H. The QTL *QPh.ISO-SAT-2* (12,265,667 bp) was co-located with the genomic region of the QTL *Rphq15*^43^. The QTL *QPh.ISO-SAT-4* (560,082,939 bp) aligned with the SNP marker JHI-Hv50k-2016-431728 (557,773,626 bp) detected in SR against *Ph* in the association mapping panel AM2017^45^. On chromosome 7H, only one QTL (*QPh.ISO-MRC-6*) associated to SR was identified at 549,976,599 bp, and it is considered as novel.

Among the 49 QTL associated with *Ph* resistance in case of APR, 17 QTL were aligned to known *Ph* loci and 34 QTL were novel. Of the five QTL detected on the chromosome 1H, the QTL *QPh.SAT2018-1* (475,085,011–475,371,901 bp) associated with *Ph* resistance in SAT2018 was in the vicinity of the previously reported SNP marker JHI-Hv50k-2016-41795 (475,643,422 bp)^45^. In addition, *QPh.SAT2019-3* (515,112,071 bp) was found in the same loci associated with the marker 11_20509 (514,738,633 bp)^52^.

No QTL was found on chromosome 2H in SAT2017. However, SAT2018 revealed three QTL, SAT2019 detected four QTL, and GUICH2018 also identified four QTL on the chromosome 2H. The QTL *QPh.SAT2018-4* (649,502,617 bp) has been reported to be involved in *P. hordei* resistance in previous study^48^. The QTL *QPh.SAT2019-5* (593,971,328–593,922,554 bp) was co-located with a previously reported QTL^45^.

On chromosome 3H, three QTL (*QPh.SAT2018-5, QPh.SAT2018-6, and QPh.SAT2018-7*) were associated with APR SAT2017, the QTL *QPh.SAT2018-6* (634,928,088) was located in the vicinity of the marker Bmag606^49^. Two QTL *QPh.SAT2019-8*, and *QPh.GUICH2018-6* associated with the APR SAT2019 and GUICH2018, respectively, were considered as novel in addition to eleven other QTL detected on 4H.

The chromosome 5H harbors eleven QTL associated with LR resistance at APR. The QTL *QPh.SAT2017-2* (528,058,903 bp) detected at SAT2017 overlapped with the QTL *Rph9.i*^44^. The QTL *QPh.SAT2018-15* (394,402,295–394,808,984 bp) was located in the vicinity of the QTL *Rph2.t*^44^. Three QTL *QPh.SAT2018-17* (530,890,859 bp), *QPh.SAT2018-18* (536,277,945 bp), *QPh.SAT2018-19* (545,187,485 bp) associated with the APR SAT2018 were previously reported in several studies^47,50,51^. On chromosome 6H, four SNP markers were significantly associated with LR resistance. The QTL *QPh.SAT2019-13* (273,286,262 bp) associated with APR to *P. hordei* was mapped in the proximity to the SNP marker bPb-3722^46^. The APR QTL *QPh.GUICH2018-10* (41,559,241 bp) detected in GUICH2018 overlapped with several QTL/genes including *Rphq3*, *QTL_Castro*, *RphQ11*, *QTL_Hickey*, and *Rph24*^16,18,46,49,53^. It also aligned with *QRph-6H.49*^54^. On the chromosome 7H, the QTL *QPh.SAT2017-4* (613,938,104 bp) was reported in many previous studies^44–47^. The genomic region in the vicinity of the QTL *QPh.SAT2019-14* (596,652,222 bp), *QPh.7H-3*, *Rphq9*, QTL_Castro, and JHI-Hv50k-2016-501477 has been reported in different studies^48,49,53^.

Two common QTL associated with *P. hordei* were detected in both SR and APR. One QTL was identified on 2H (at 643,275,772–644,195,113 bp), and the other one QTL on chromosome 7H (548,075,313–549,976,599 bp). These QTL were considered novel. The highly significant QTL *QPh.ISO-MRC-5* (644,195,113 bp), with the lowest p-value of 8.852 × 10^–05^ and the highest R^2^ of 7.541% detected for the *Ph* isolate ISO-MRC overlapped with the APR QTL *QPh.SAT2018-3* (643,275,772 bp) at SAT2018.

Understanding the disease resistance mechanism of the putative candidate genes (CGs) associated with SNP markers can help in developing functional and predictive markers. Based on functional annotation, many of the identified CGs were involved in plant defense mechanisms, suggesting that they may contribute to LR resistance. The major *R*-genes encode the NLR proteins, harboring the central nucleotide-binding domain (NBS) and a C-terminal leucine-rich repeat (LRR) domain. The NLR proteins are intracellular immune receptors that recognize pathogen effectors (dominant avirulence gene product) via direct or indirect interaction in a gene-for-gene interaction, causing plant cell death in the area surrounding the initial point of infection to stop the further spread of the pathogen. This response is efficient for biotrophic pathogens that require living tissue. Seven SR and twelve APR SNP markers showed homology with NLR and RLK (receptor like kinases). The CGs associated with the QTL *QPh.SAT2017-4* (7H, 613,938,104 bp) showed homology with NB-LRR disease resistance proteins (Table 4). The NLR proteins have been reported in several leaf rust resistance genes in wheat and barley such as *Lr1, Lr21, Lr22a, Lr10* and *Rph1*^61–65^. In durum wheat, an association mapping analysis showed that the wPt-8460 marker on the chromosome 2B corresponding to NBS-LRR gene was associated with stem-rust resistance^66^.

Several cell signaling components encode protein kinases. In this study, the CGs associated with the two SR QTL *QPh.ISO-MRC-1* (1H, 5,345,263 bp) and *QPh.ISO-SAT-3* (6H, 407,625,773 bp) and the two APR QTL *QPh.SAT2019-7* (2H, 630,365,638 bp) and *QPh.SAT2018-3* (2H, 643,275,772 bp), encoded proteins possessing kinase domain. Interestingly, the *Ph* resistance gene *Rph22* encodes a lectin receptor kinase, a member of a protein family located in the plasma membrane^67^. In addition, serine/threonine kinases have been implicated in resistance to many diseases including stem rust in barley (*rpg1*)^68^, *Pseudomonas syringae* in tomato (*Pti1*)^69^, powdery mildew in wheat (*Pm21*)^70^, and bacterial blast caused by *Xanthomonas oryzae* pv. *Oryzae* in rice (*Xa21*)^71^.

The putative candidate genes (CG) adjacent to the two APR QTL *QPh.SAT2019-9* (JHI-Hv50k-2016-226367; 210,117 bp) and *QPh.SAT2018-14* (JHI-Hv50k-2016-272121; 601,760,759 bp) on the chromosome 4H, and to the QTL *QPh.SAT2018-1* (JHI-Hv50k-2016-41672; 475,371,901 bp) on chromosome 1H encode an ABC transporter protein. The wheat LR resistance gene *Lr34* encodes a ABC transporter protein that is effective at the adult plant stage^72^, and it confers effective resistance to multiple biotrophic pathogens including leaf rust, stem rust, stripe rust, and powdery mildew in wheat^73^, and powdery mildew and leaf rust in barley^74^.

Overall, we identified 59 QTL associated with resistance to barley leaf rust at the seedling and the adult plant stages. In addition to the detection of already known loci, 65.6% of the QTL identified were novel, providing additional information for barley breeders searching for new sources of disease resistance and their associated markers. We were able to identify highly resistant FIGS-LR and GCP accessions with high frequency of the favorable alleles of the significant markers that can be used in barley breeding program. Further characterization and validation of QTL are needed to effectively introgress the identified LR resistance loci into elite barley germplasm via marker-assisted selection. Understanding the molecular mechanism of the identified LR resistance and CGs can help in the process of developing functional and predictive markers. It also contributes to a better understanding of the genetic mechanisms that control barley leaf rust resistance and provides the basis for their characterization, cloning, and gene manipulation in future studies.

## Methods

### Plant material

The germplasm used in this study consisted of 218 barley accessions originated from different countries. This experimental population included two subsets; 142 accessions of GCP (Generation Challenge Program) and 76 accessions of FIGS-LR (subset selected for leaf rust using the Focused Identification of Germplasm Strategy). These two barley subsets were composed of 57 two-row and 161 six-row type genotypes, including barley cultivars and landraces, provided by the genebank of the International Center for Agriculture Research in the Dry Areas (ICARDA), Rabat, Morocco. This germplasm collection was evaluated for seedling resistance using two *P. hordei* isolates (ISO-MRC and ISO-SAT) and for adult plant resistance under field conditions in four environments^23^. The full list of barley accessions and their detailed infection response to *P. hordei* at both stages has been presented in Supplementary Table S1.

### Phenotyping for seedling resistance to *P. hordei*

Barley accessions of both subsets were screened for the seedling resistance with two Moroccan *P. hordei* isolates (ISO-MRC and ISO-SAT) selected based to their wide virulence spectra across the 19 Bowman near-isogenic lines (NIL) of barley differentials with known *Rph* genes. ISO-MRC was virulent on NILs carrying *Rph2.b, Rph3.c, Rph4.d, Rph5.e, Rph6.f Rph5, Rph7.g, Rph8.h, Rph9.i, Rph10.o, Rph11.p, Rph9.z Rph12, Rph2.j, Rph2.y, Rph2.t*, whereas the isolate ISO-SAT was virulent on NILs carrying *Rph1.a, Rph3.c, Rph4.d, Rph8.h, Rph9*.

4–5 grains of each genotype were grown in plastic cone (3.8 cm in diameter and 14 cm in length) arranged in 14 × 7-unit tray (98 genotypes/tray). Barley genotypes Philadelphia and Lakhan were used as resistant and susceptible checks, respectively. At the first leaf stage (10–12 days), a uniform inoculation was conducted by spraying 15 mg of urediniospores suspended in 10 ml of light mineral oil (Novec 7100, Sigma Aldrich) per tray using an airbrush (Revell, München, Germany). Inoculated plants were left at the room temperature for 20 min for allowing the oil evaporation and were fine-misted with water then moved to the growth chamber in the dark for 24 h at 18 °C with relative humidity close to 100%. Seedlings were kept in the growth chamber at 20 °C with 16h light/8h dark photoperiod for disease development. LR infection types were recorded 10–12 days post-inoculation based on the following scale^75^: immune (0), resistant (0; and 1), moderately resistant (2), moderately susceptible (3), or susceptible (4).

### Phenotyping for adult plant resistance to *P. hordei*

The APR experiments were conducted in the fields at the INRA experimental station of Sidi Allal Tazi (34° 52ʹ N, 6.32175 W) during 2016–2017 (SAT2017), 2017–2018 (SAT2018), 2018–2019 (SAT2019), and at GUICH (33° 58ʹ 59.7ʹʹ N 6° 51ʹ 41.6ʹʹ W) during 2017–2018 (GUICH2018).

Field plots were planted in single rows of 1m with 0.5 m row spacing between adjacent accessions in an augmented block design. The seed mixture of susceptible cultivars Bowman and Aglou were grown in a long single row as spreader row after each block. Natural infection was established at SAT, but at GUICH LR epidemic was initiated by spraying the susceptible spreader rows with LR inoculation as described in our previous study^23^. Briefly, two spray inoculations were made during evening hours with urediniospores suspension (1g of urediniospores in 200 ml of mineral oil) on the trial using an airbrush (Revell, Munchen, Germany). The establishment and spread of the disease were favored by covering the spreader rows with a plastic sheet overnight and by periodic sprinkler irrigation. LR severity for GCP and FIGS_LR subsets was recorded at Zadoks growth stage GS 65–77^76^ according to the modified Cobb’s scale^77^ which includes disease severity (percentage of leaf area covered with rust urediniospores) as well as infection type. The infection types were recorded as 0 (Immune), no visible infection on plants; R (resistant), visible chlorosis or necrosis, no uredia are present; MR (moderately resistant), small uredia are present and surrounded by either chlorotic or necrotic areas; MS (moderately susceptible), medium sized uredia are present and possibly surrounded by chlorotic areas; S (susceptible), large uredia are present, generally with little or no chlorosis and no necrosis.

The field disease severity data was converted to Coefficient of Infection (CI) by multiplying the percent disease severity (0–100%) by the constant values of infection types (R = 0.2, MR = 0.4, MS = 0.8, S = 1)^78^, and the accessions were rated based on the average coefficient of infection (ACI) where values of 0–7, 8–16, 17–29, 30–50, and > 50 were considered as resistant, moderately resistant, moderately susceptible, susceptible, and highly susceptible, respectively.

### Genotyping and quality control

DNA from the 218 barley accessions of the FIGS-LR and GCP subsets was extracted from the leaves of two weeks old seedlings, and subsequently genotyped using the Illumina iSelect 50K SNP array for barley (Illumina, San Diego, CA, USA)^79^ at the Cereal Crop Research Unit, USDA-ARS Genotyping Laboratory Fargo, North Dakota, USA^80^. A total of 36,864 markers were scored, and after the quality control, 32,686 SNPs were retained with missing data of less than 20% and minor allele frequency MAF > 5%. Missing data imputation was performed using the LDKNNimp method^81^ implemented in TASSEL version 5.2^82^, and the final filtered data was then used for further genetic analysis. Genome-wide SNP marker density of 32,686 SNP markers used in this study was plotted using the CMplot R package (https://github.com/YinLiLin/R-CMplot).

### Genetic diversity analysis

Genetic diversity including polymorphism information content (PIC) and gene diversity (GD) were estimated for FIGS_LR and GCP subsets separately and in combination using PowerMarker V3.25^83^. Minor allele frequency (MAF) was calculated using PLINK (–freq).

The degree of linkage between nearby loci was characterized using linkage disequilibrium (LD) analysis in PLINK 1.9^84^. The LD was assessed by calculating the Pearson correlation values (*R*^2^) for all pairs of SNPs located at a maximum of 5000kb. *R*^2^ and pairwise distance between SNPs were used to generate LD decay plots using ggplot2 R package. The LD decay was calculated when the *r*^2^ value decreased below a threshold level (*R*^2^ < 0.2).

### Population structure

Population structure of the 218 barley genotypes was assessed using molecular marker information with sparse non-negative matrix factorization (SNMF)^85^ implemented in the R package ‘landscape and ecological association (LEA)’^86^. For this analysis, the cross-entropy was calculated for each K number of clusters ranging from K = 1 to K = 10, with ten replicates for each K value. The K with the lowest cross-entropy was selected as the optimal number of clusters. The ancestry proportion matrix was calculated for K = 3. The PCA and clustering groups for barley accessions was plotted using the R package ggplot2^87^. The kinship matrix analysis^88^ in TASSEL 5.2.82^82^ was conducted to define the degree of genetic covariance between pairs of given accessions. It was estimated using the complete set of markers that passed the quality filtering based on the scaled IBS method.

### Genome-wide association analysis

The Genome wide association study (GWAS) was performed using GAPIT3 package in R^89^ with 32,686 filtered SNP markers and *P. hordei* responses at the seedling and at the adult plant stages. Different statistical models were used to investigate the best fitting model for the present study including: the general linear models (GLM), mixed linear model (MLM), settlement of MLM under progressively exclusive relationship (SUPER), multiple-locus MLM (MLMM), fixed and random model circulating probability unification (FarmCPU), and Bayesian-information and linkage-disequilibrium iteratively nested keyway (BLINK). Finally, the MLM (PCA + K) model was used for GWAS analysis in this study as it was the best fitting model. Marker alleles with *p* ≤ 0.0005(− log10 *p* ≥ 3.3) were declared significantly associated with LR resistance as previously described by other researchers. A False discovery rate (FDR) of q < 0.05 was used to determine significant QTLfor multiple comparisons^90^. In the output, *R*^*2*^ values were used to represent the proportion of the phenotypic variation explained by each marker. The sign of the allelic effect estimate is determined by the nucleotide that ranks second in alphabetical order. For example, if the nucleotides at a SNP are “A” and “T”, then a positive allelic effect (susceptibility) indicates that “T” is favorable. The *p*-values from different models were used to generate Manhattan and quantile–quantile (QQ) plots using the qqman R package^91^.

### QTL alignment and candidate genes

Marker sequences that revealed significant associations with resistance to barley leaf rust were annotated for putative candidate genes (CGs) and their function using BARLEX database (https://apex.ipk-gatersleben.de/apex/f?p=284:10; accessed on 2 March 2022) and Barleymap pipeline^92^. Perfect match or homologous sequences were identified based on 100% query coverage, an Expect (E) value (0–1^40^), and identity higher than 99%. Potential CGs were considered based on their functional domains involved in plant disease resistance.

To align QTL detected for *P. hordei* resistance in this study with QTL reported in previous association mapping studies, we used GrainGenes (https://wheat.pw.usda.gov/GG3/) database to find a strongly linked marker/sequence information associated with published QTL, and then align them on Morex V3^93^ using Barleymap pipeline^92^.

### Supplementary Information

Supplementary Information.

## Data Availability

All data generated or analyzed during this study are present in this paper and the Supplementary Materials.
