# Supplementary material for: Genome wide association study of seedling and adult plant leaf rust resistance in two subsets of barley genetic resources
Source: Sci Rep. 2024 Jul 4;14:15428. doi: 10.1038/s41598-024-53149-2 (PMC11224298; doi:10.1038/s41598-024-53149-2)
Supplement: Supplementary file 1 — Supplementary Information. [file 41598_2024_53149_MOESM1_ESM.docx]

Genome Wide Association Study of Seedling and Adult Plant Leaf Rust Resistance in two subsets of barley genetic resources

Mariam Amouzoune^1, 2*^, Sajid Rehman^2,3^, Rachid Benkirane^1^, Sripada Udupa^2^, Sujan Mamidi^4^, Zakaria Kehel^2^, Muamer Al-Jaboobi^2^, Ahmed Amri^2^


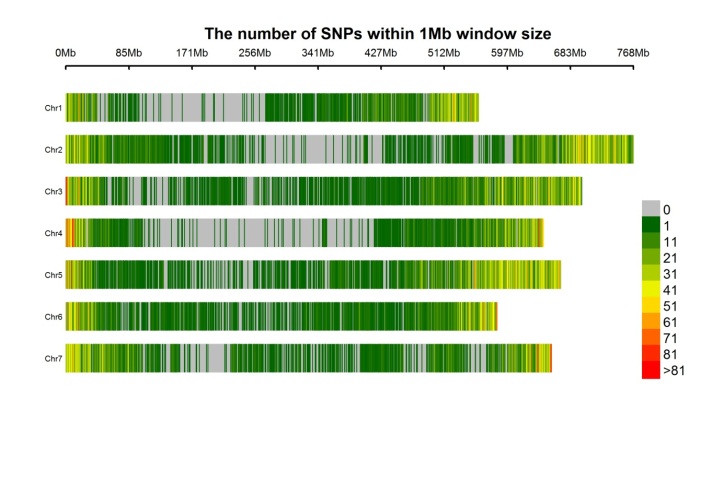


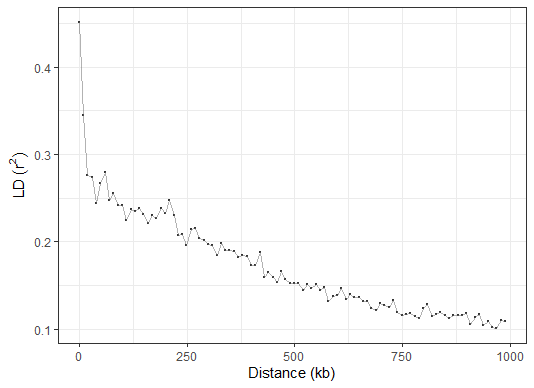


# Supplementary Figure S1. SNP marker density plot per chromosome of 32,686 SNPs of both FIGS-LR and GCP subsets based on their physical position. The horizontal axis shows the length of the chromosome (Mb). Color gradient scale on the right indicates the SNP density.

**Supplementary Figure S2.** Linkage disequilibrium (LD) decay in 218 barley genotypes of FIGS-LR and GCP subsets by plotting r^2^ values against physical position (kb).

**Supplementary Figure S3.** QQ plots for LR resistance at the seedling (ISO-MRC, ISO-SAT) and the adult plant stages (SAT2017, SAT2018, SAT2019, and GUICH2018) using all GAPIT models in both barley subsets FIGS-LR and GCP.


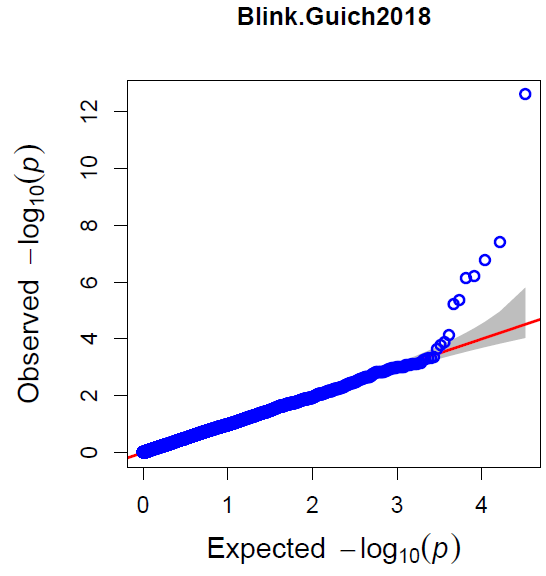

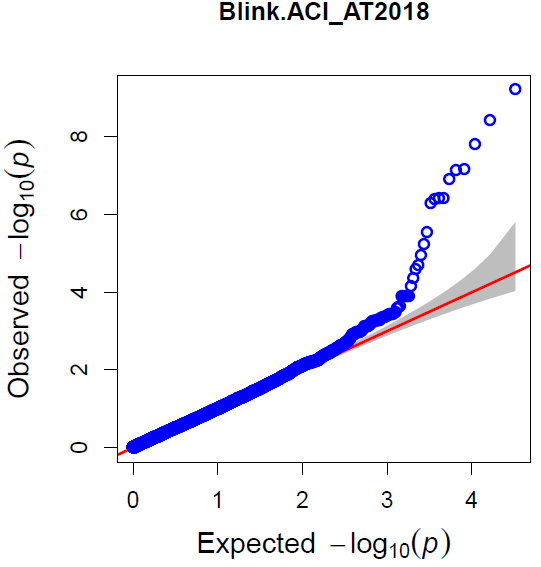

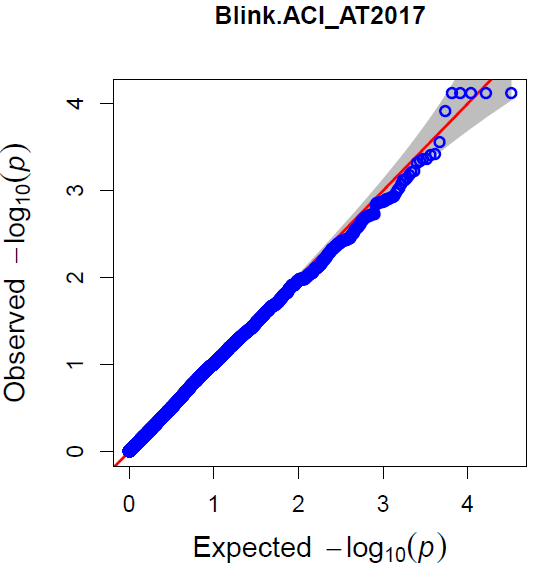

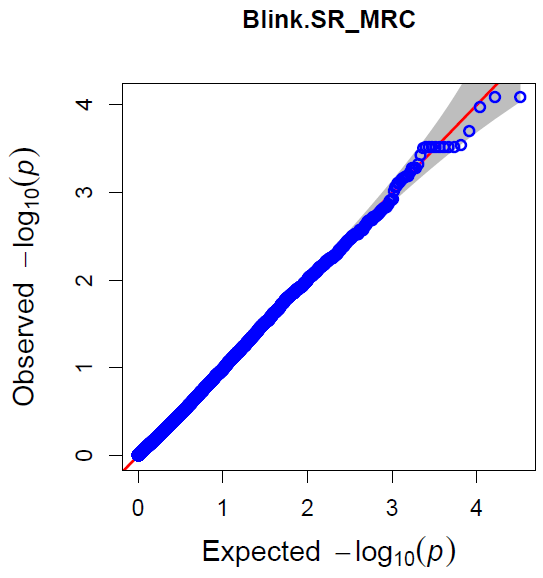

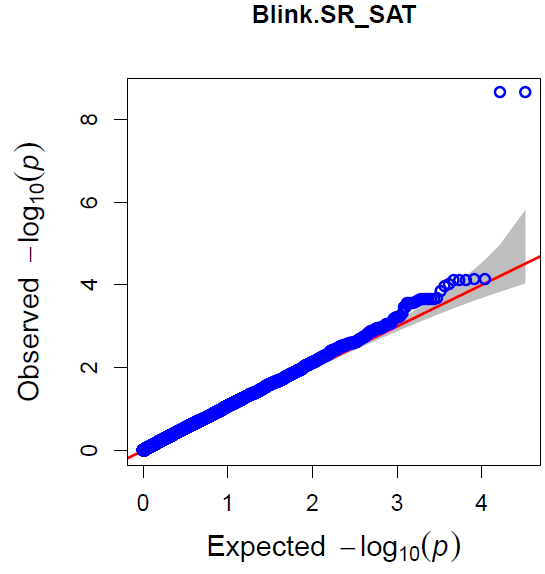

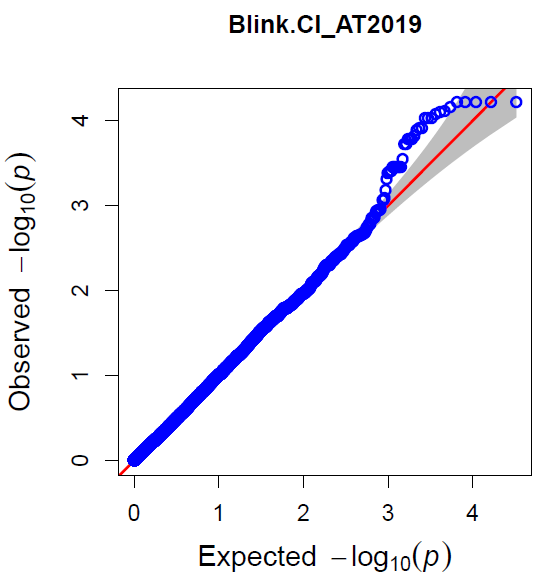

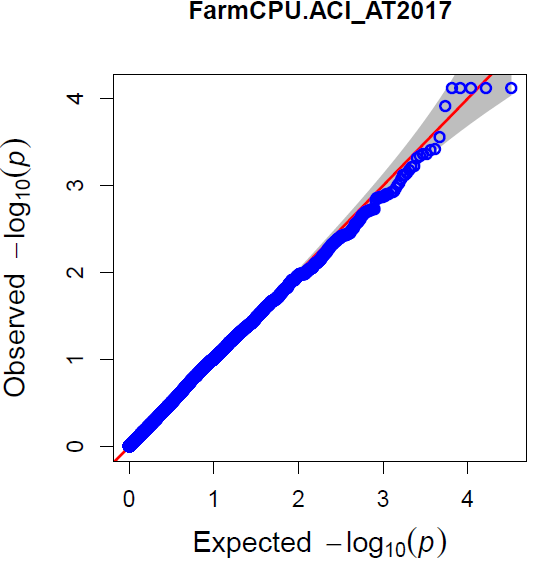

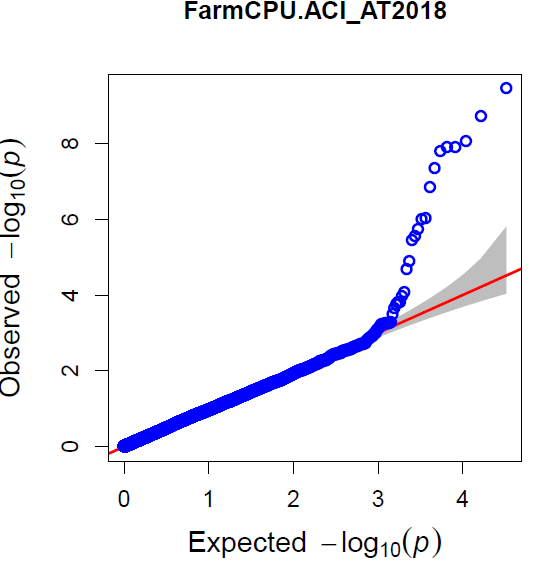

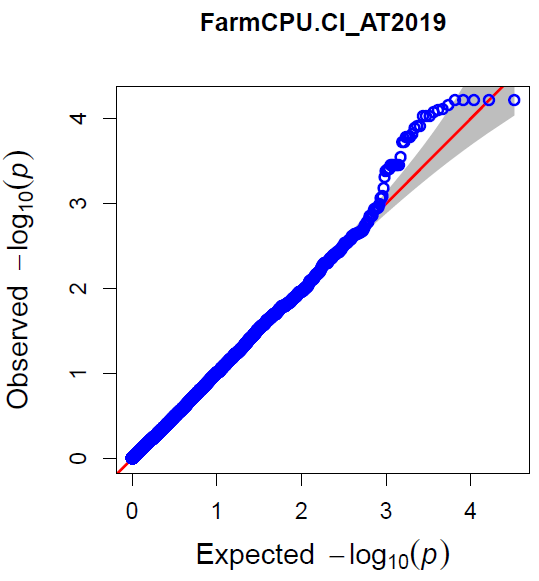

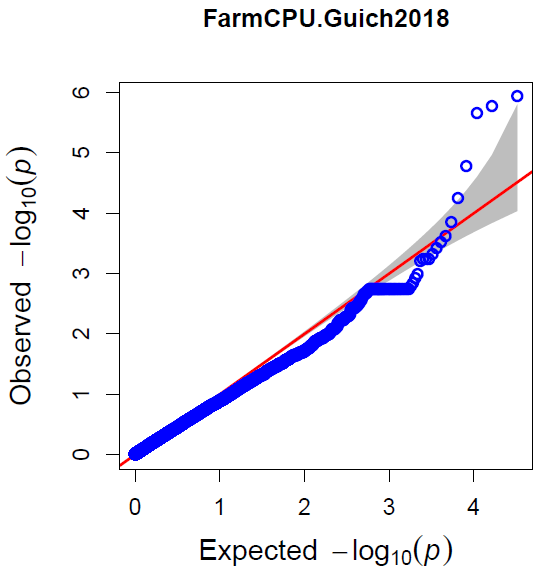

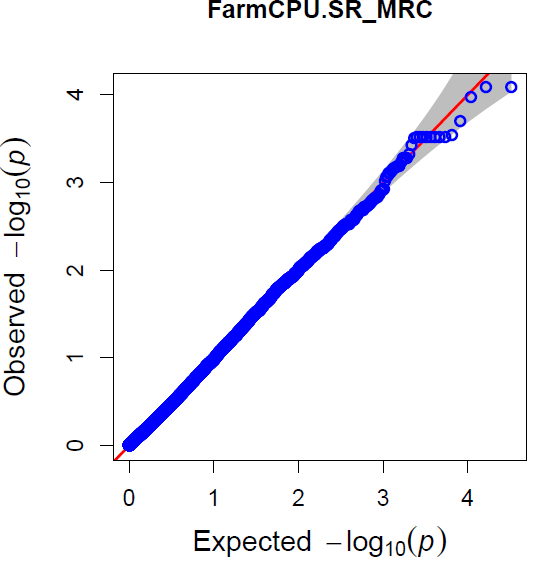

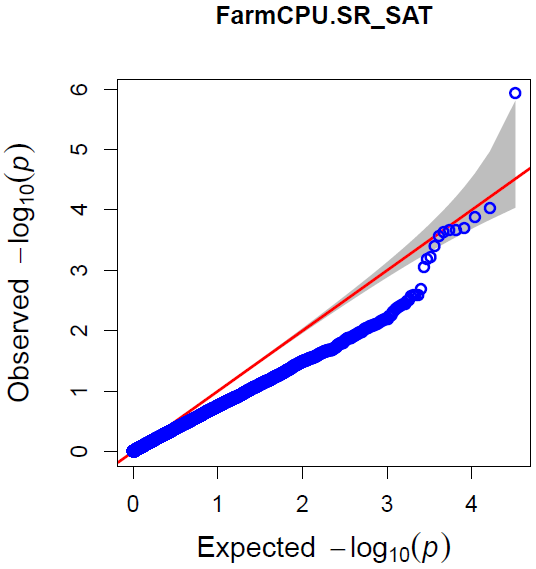

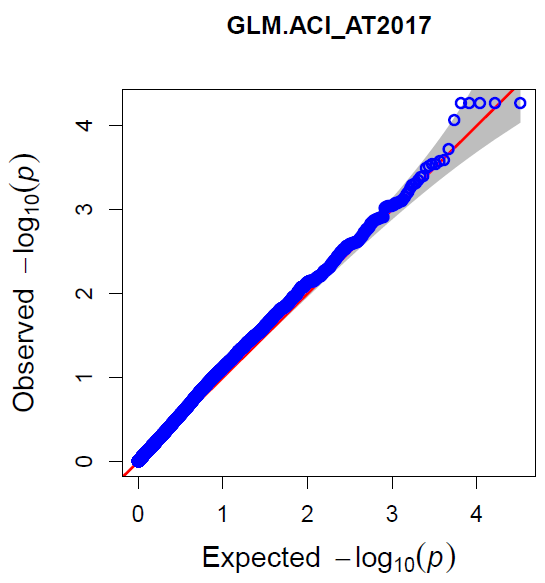

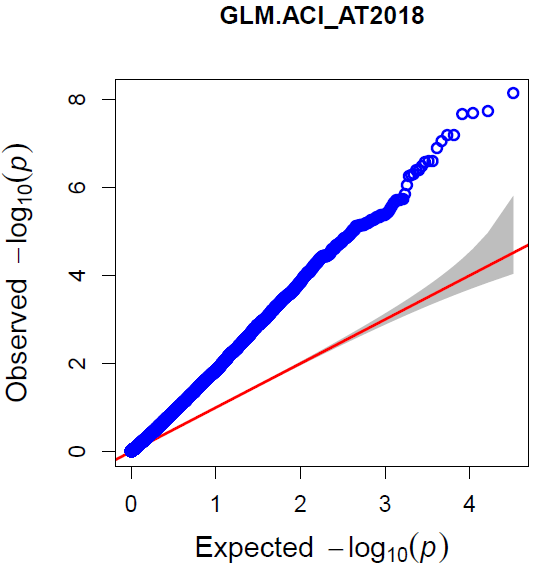

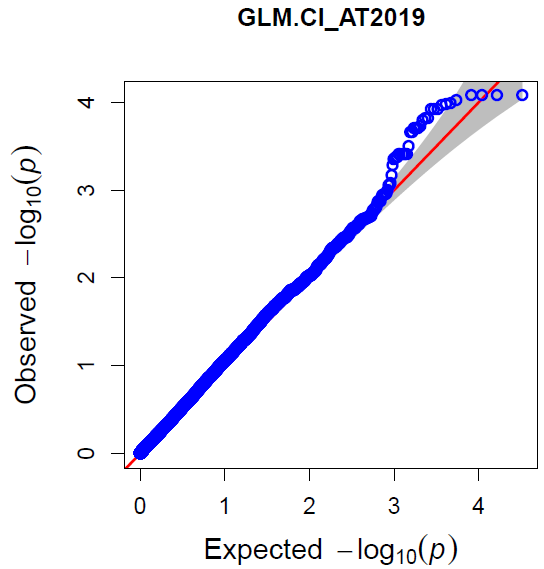

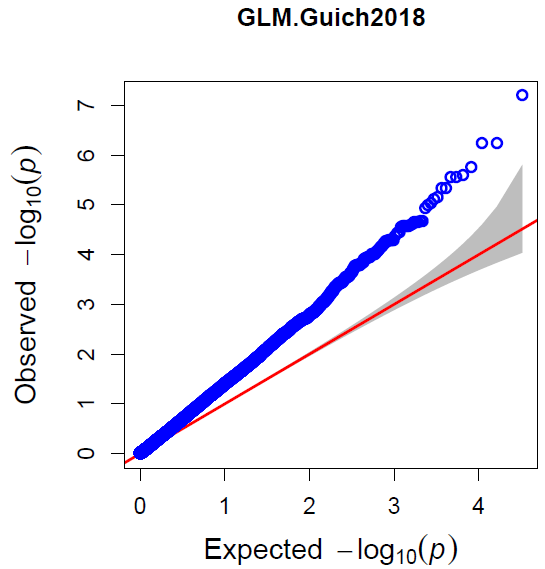

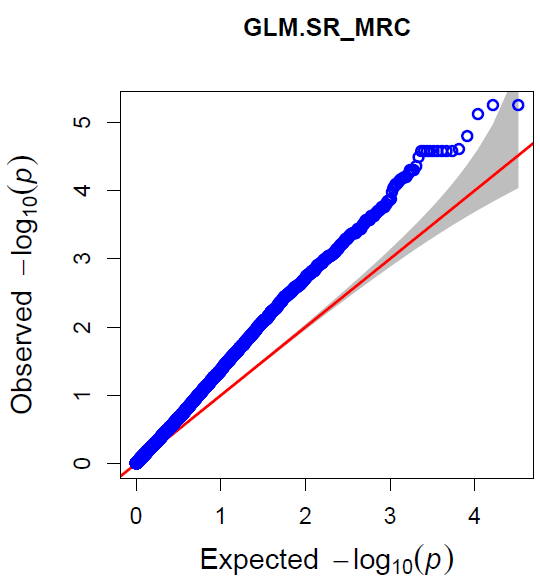

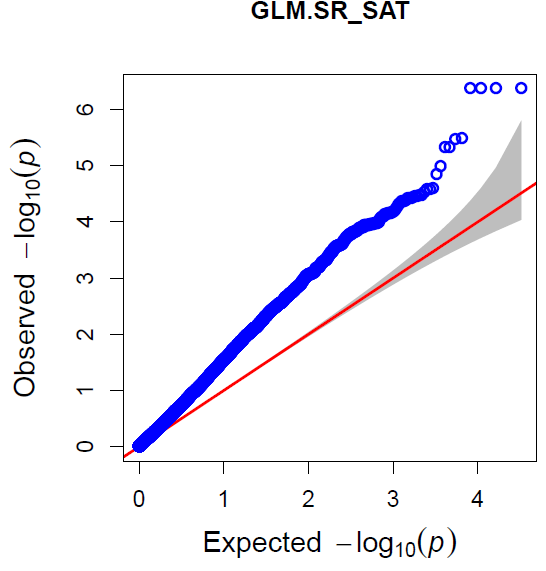

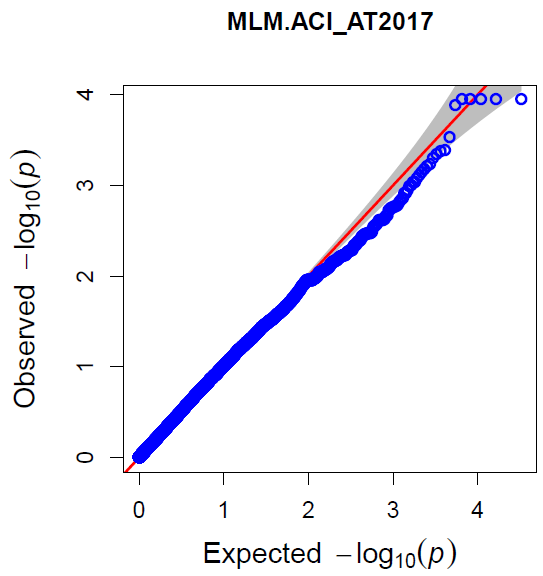

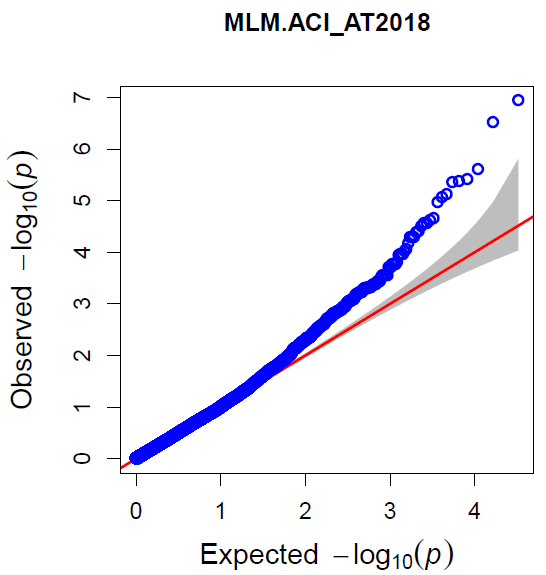

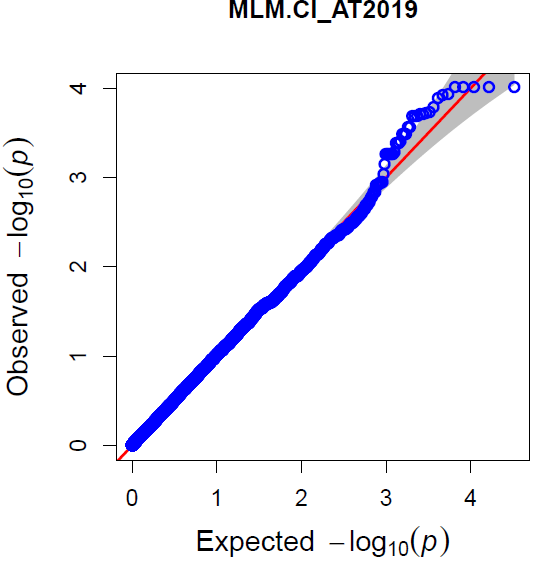

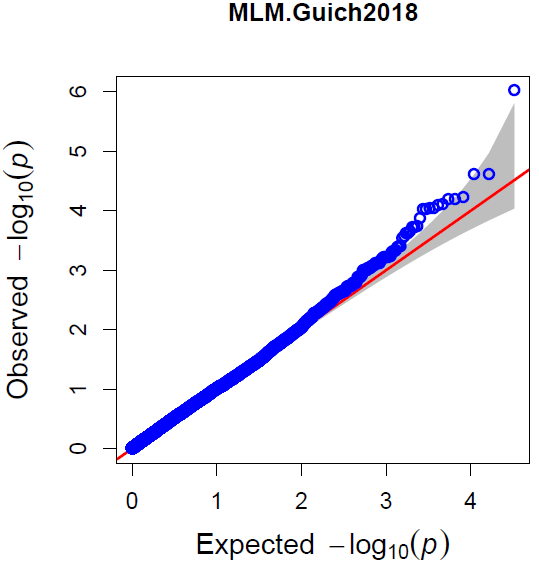

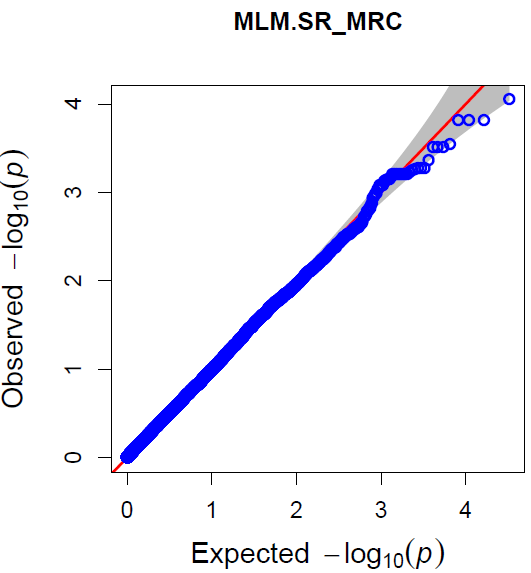

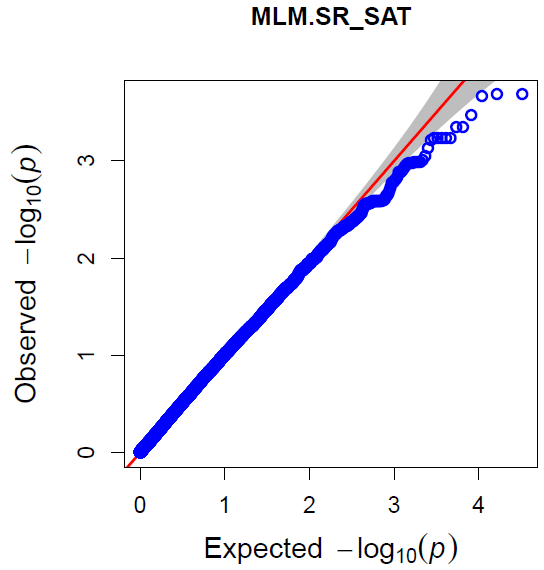

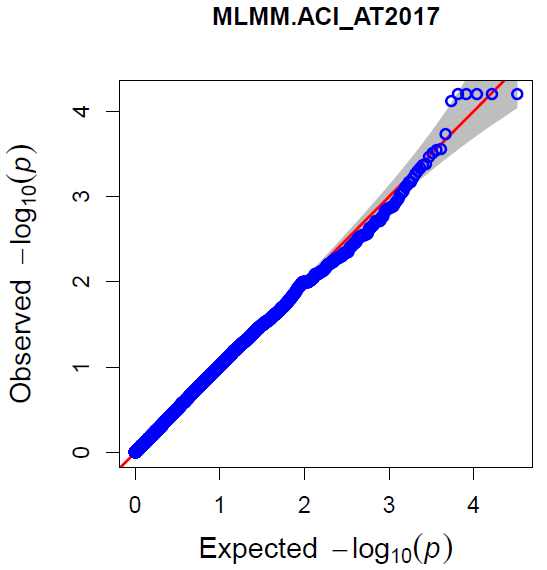

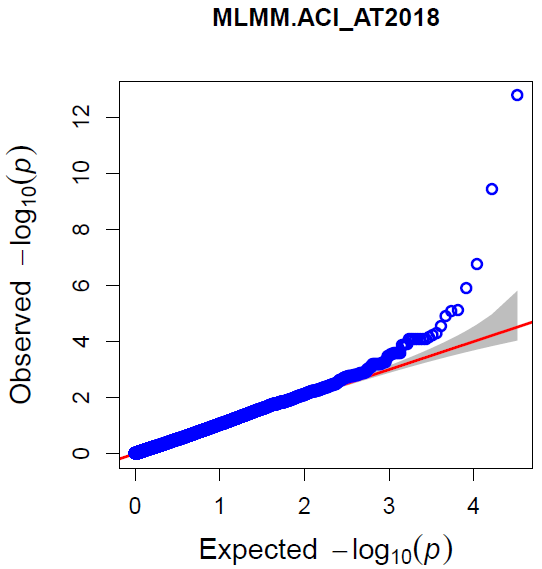

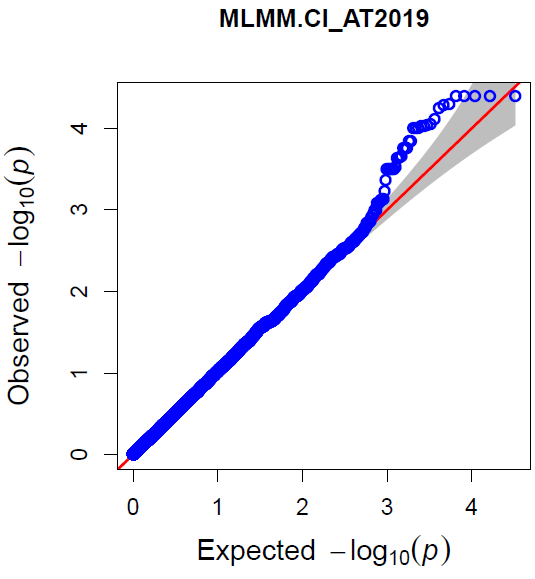

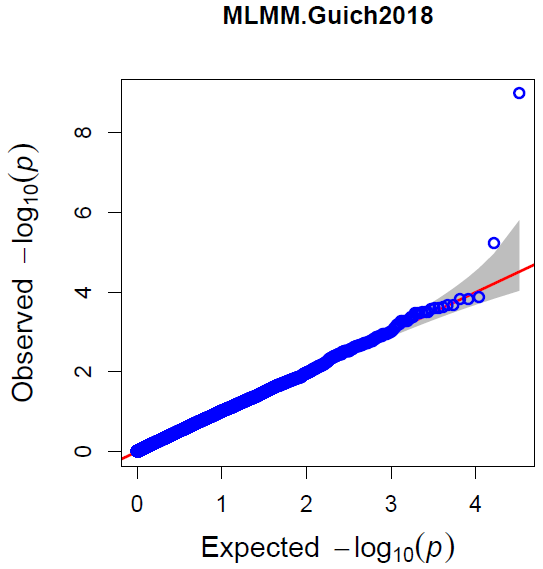

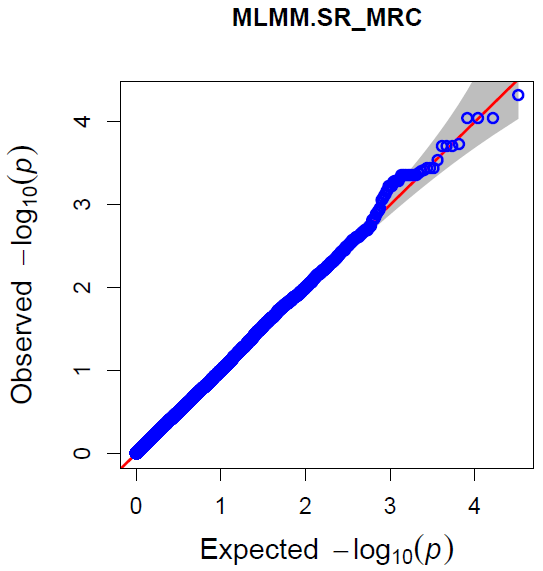

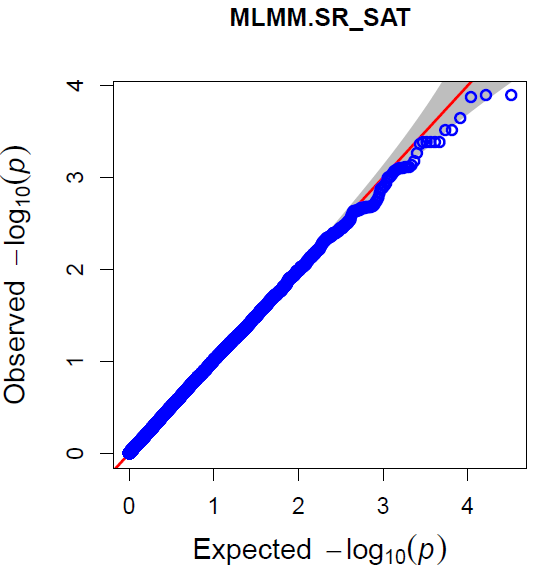

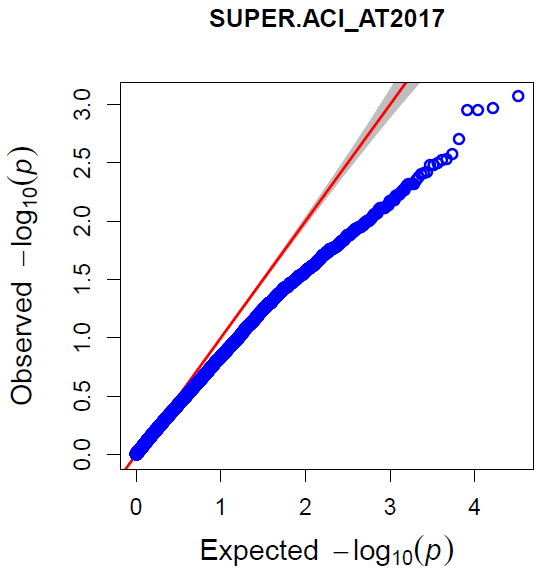

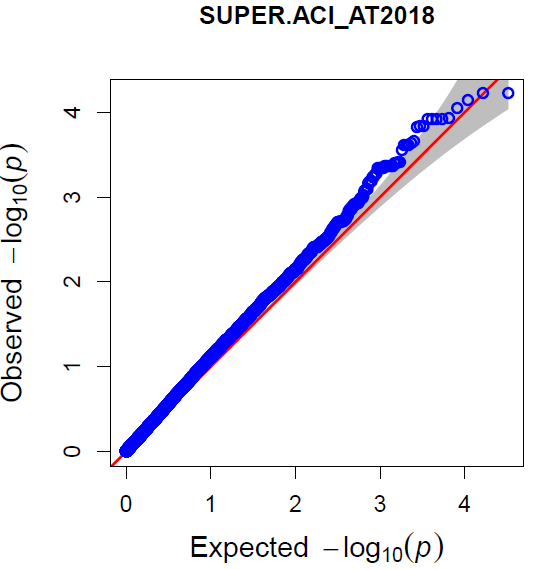

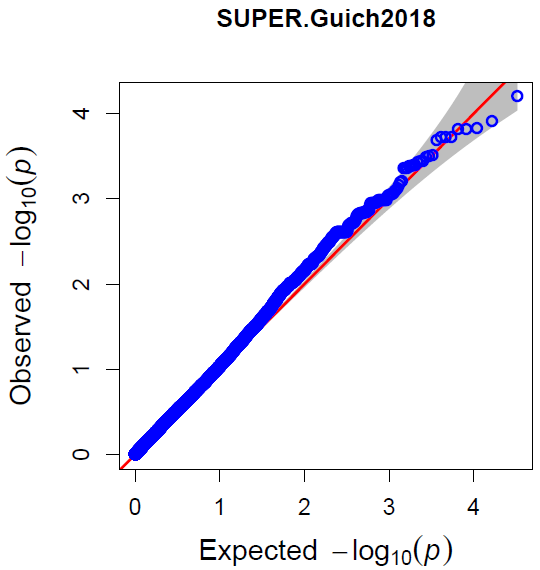

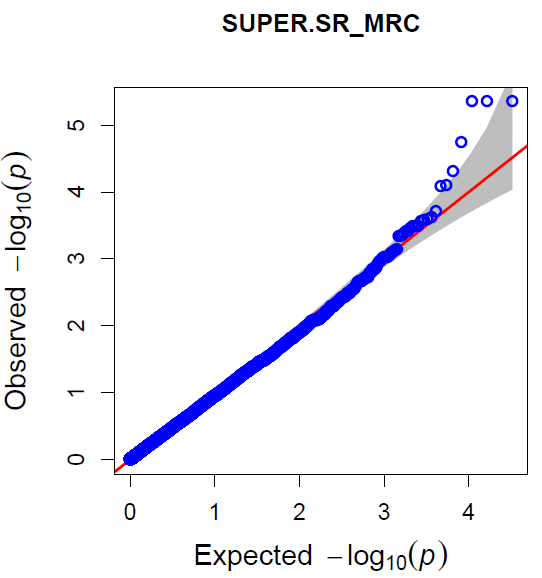

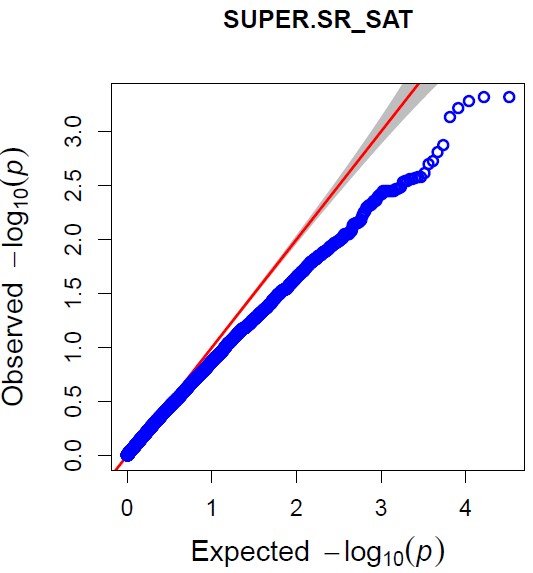

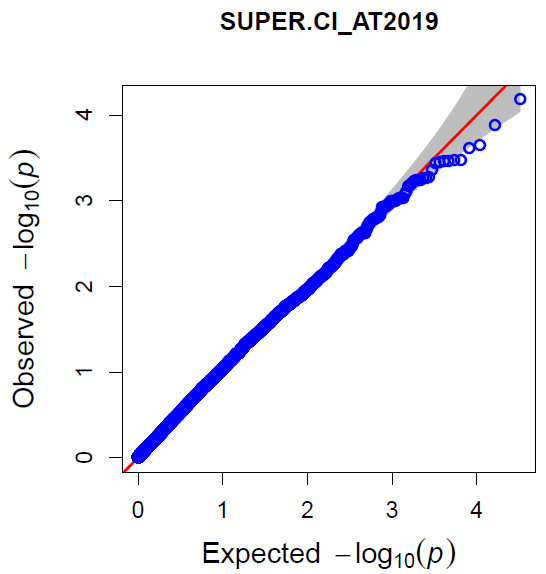


**Supplementary Figure S4.** Genome-wide association mapping of barley leaf rust resistance at the seedling (ISO-MRC, ISO-SAT) and adult plant stages (SAT2017, SAT2018, SAT2019, and GUICH2018) using the MLM (PCA+K) model in GAPIT3. (**a**) Quantile-Quantile (Q-Q) plots of marker-trait association; (**b**) Manhattan plots shows –log10 of p-values from genome-wide association mapping against the positions of SNPs on all chromosomes of both barley subsets (FIGS-LR and GCP). The p-value threshold (-log10 p-value (0.0005) ≥ 3.3) was used to declare significant QTL.

**a)**

**b)**


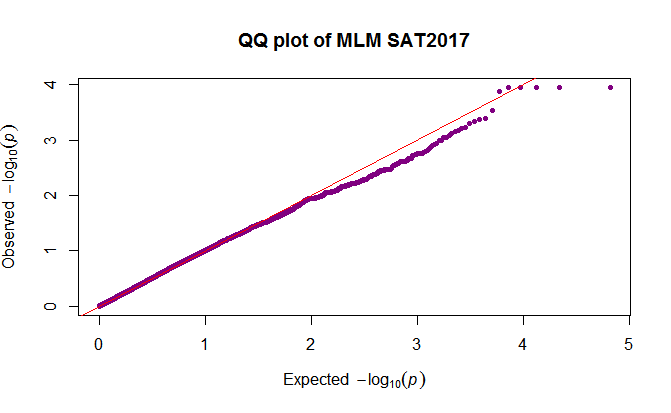

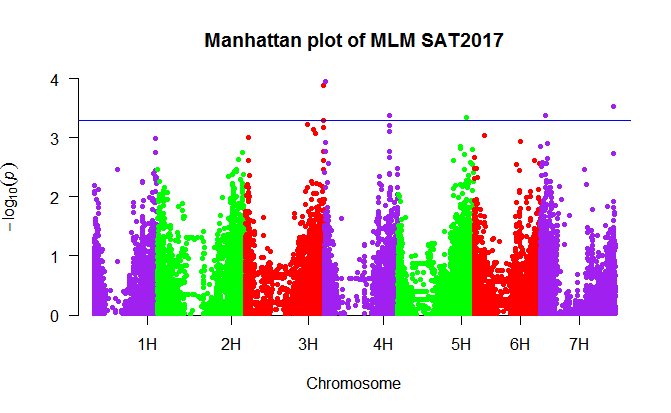

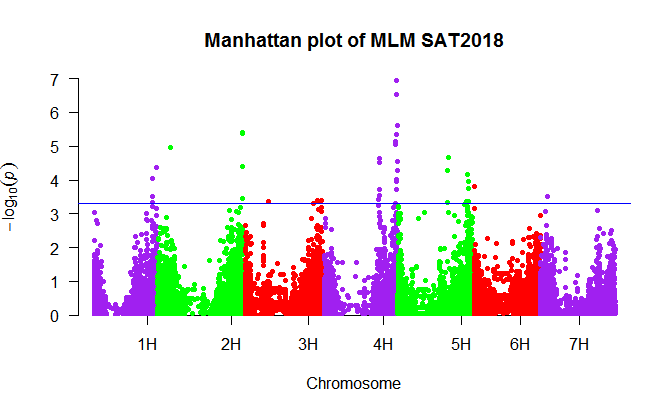

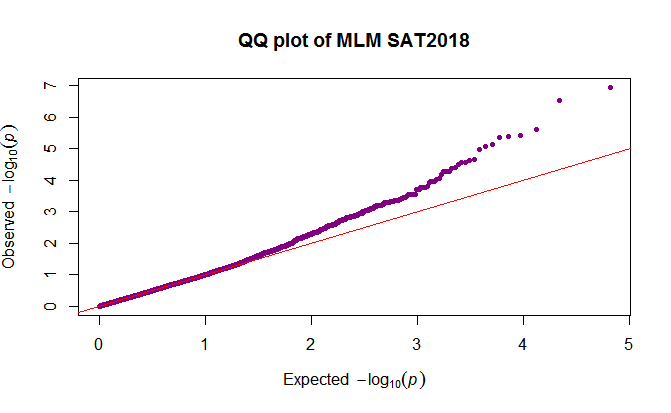

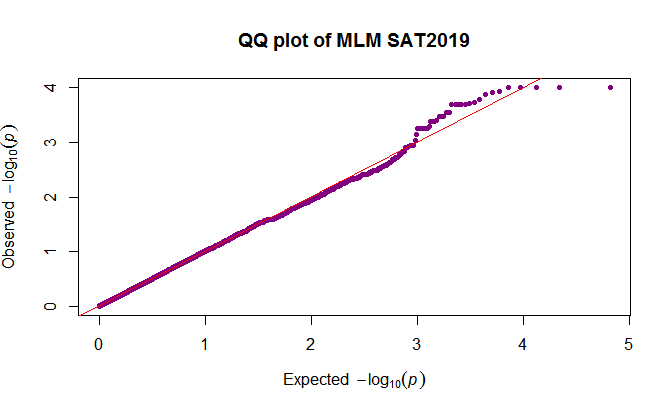

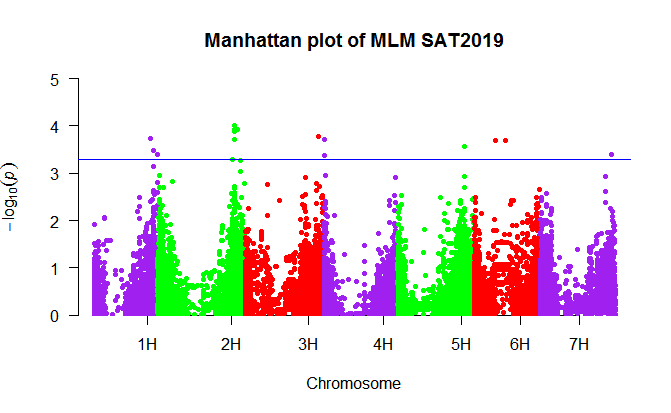

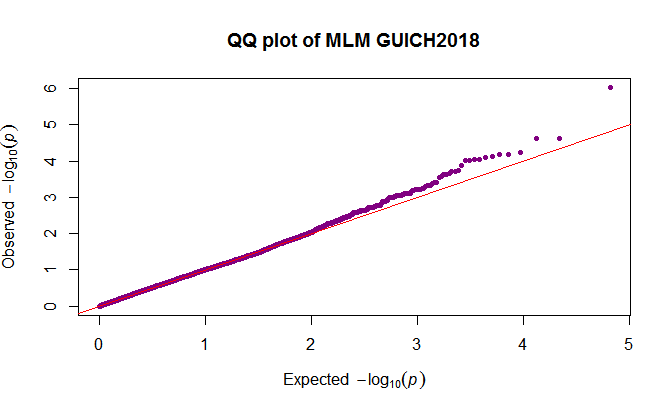

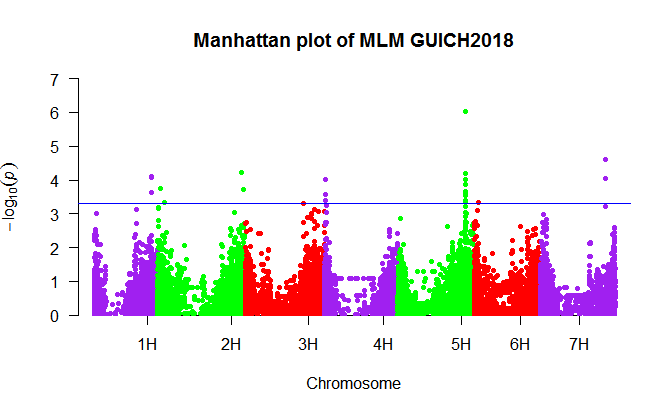

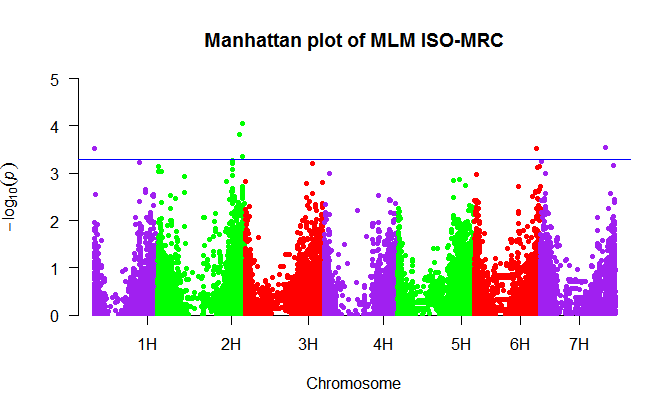

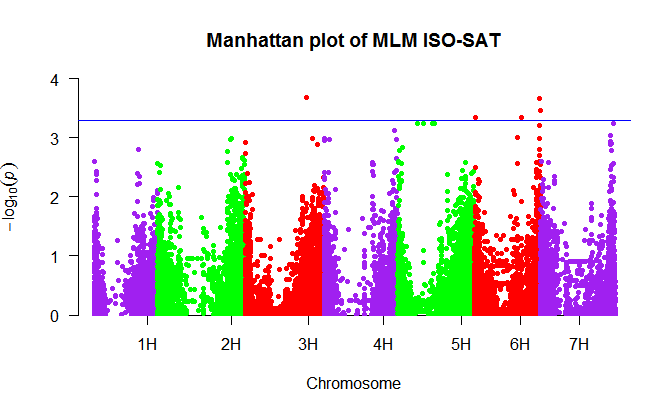

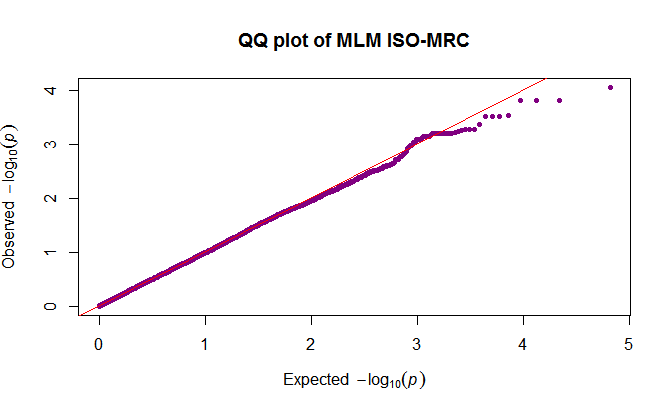

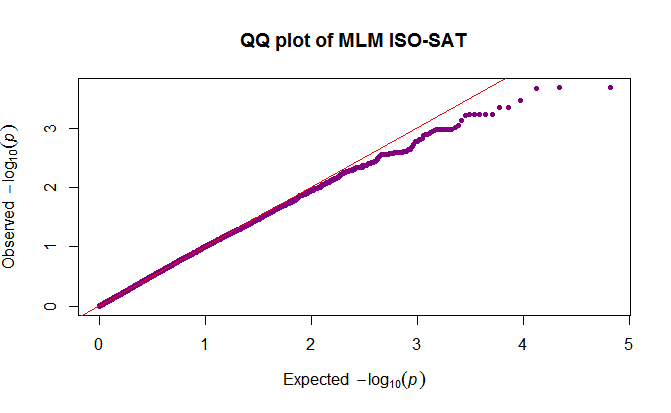


Supplementary Table S1: Evaluation of the two barley subsets (FIGS-LR and GCP) for the resistance *to P. hordei* at the seedling and at adult plant stages.

| **IG** | **Subset** | **RT** | **Origin** | **ISO-SAT** | **ISO-MRC** | **SAT2017** | **SAT2018** | **GUICH**  **2018** | **SAT2019** |
| --- | --- | --- | --- | --- | --- | --- | --- | --- | --- |
| 16875 | FIGS-LR | 6 | RUS | 2 | 1 | 47 | 0.3 | 3.2 | 10 |
| 16880 | FIGS-LR | 6 | RUS | 2 | 3 | 36 | 2 | 0.2 | NA |
| 16883 | FIGS-LR | 6 | RUS | 2 | 2 | 27 | 2.1 | 26 | NA |
| 17396 | FIGS-LR | 6 | MNE | 2 | 1 | 53 | 0.4 | 7.23 | NA |
| 17398 | FIGS-LR | 6 | MNE | 3 | 3 | 20 | 0.4 | 0.4 | NA |
| 17424 | FIGS-LR | 2 | MNE | 2 | 2 | 24 | 2 | 27.2 | NA |
| 18755 | FIGS-LR | 6 | TUR | 2 | 2 | 51 | 0.3 | 2.2 | NA |
| 18798 | FIGS-LR | 6 | TUR | 2 | 2 | 15 | 0.5 | 3.55 | 20 |
| 18938 | FIGS-LR | 6 | GRC | 2 | 2 | 3 | 0.2 | 2.2 | 30 |
| 18953 | FIGS-LR | 6 | GRC | 1 | 2 | 34.5 | NA | 8 | 6 |
| 18955 | FIGS-LR | 6 | GRC | 1 | 2 | 28 | 0.2 | 11.1 | 30 |
| 19037 | FIGS-LR | 6 | GRC | 1 | 2 | 16 | 0.5 | 1.25 | NA |
| 19095 | FIGS-LR | 6 | GRC | 2 | 2 | 8.1 | 0.25 | NA | NA |
| 19209 | FIGS-LR | 6 | TUR | 2 | 2 | 16 | 1.7 | 5.45 | 10 |
| 19617 | FIGS-LR | 6 | GRC | 2 | 2 | 3.2 | 0.3 | 10.15 | NA |
| 21781 | FIGS-LR | 6 | ETH | 4 | 2 | 40.1 | NA | 2.95 | 30 |
| 21867 | FIGS-LR | 2 | ETH | 3 | 3 | 40 | 48 | 2.2 | 6 |
| 21976 | FIGS-LR | 2 | ETH | 4 | 2 | 45 | 60 | 5.3 | 18 |
| 21978 | FIGS-LR | 6 | ETH | 3 | 3 | 50 | 12 | 3.8 | NA |
| 22046 | FIGS-LR | 2 | ETH | 2 | 2 | 4 | 60 | 0.4 | NA |
| 22319 | FIGS-LR | 6 | ETH | 3 | 2 | 70 | 45 | 12.45 | 8 |
| 22488 | FIGS-LR | 2 | ETH | 4 | 2 | 70 | 60 | 0.73 | NA |
| 22489 | FIGS-LR | 6 | ETH | 2 | 2 | 65 | 40 | 7.45 | 27 |
| 22496 | FIGS-LR | 2 | ETH | 3 | 2 | 60 | 40 | 5.55 | NA |
| 22503 | FIGS-LR | 6 | ETH | 3 | 2 | 24 | 60 | 6.7 | NA |
| 22539 | FIGS-LR | 6 | ETH | 3 | 2 | 53 | 50 | NA | NA |
| 22848 | FIGS-LR | 2 | ETH | 3 | 2 | 61 | 12 | 18.6 | NA |
| 23042 | FIGS-LR | 6 | ETH | 3 | 2 | 52 | 60 | NA | 16 |
| 23112 | FIGS-LR | 2 | ETH | 3 | 2 | 60 | 40 | 6.5 | NA |
| 23425 | FIGS-LR | 6 | ETH | 3 | 2 | 70 | 30 | 13.5 | 30 |
| 23584 | FIGS-LR | 6 | MKD | 3 | 3 | 20 | 0.4 | 9 | NA |
| 23597 | FIGS-LR | 6 | MKD | 4 | 3 | 9 | 3.2 | 1.3 | 10 |
| 25306 | FIGS-LR | 6 | UKR | 2 | 1 | 14 | NA | NA | 16 |
| 25530 | FIGS-LR | 6 | PSE | 2 | 1 | 20 | NA | 7.15 | NA |
| 28367 | FIGS-LR | 6 | IND | 3 | 2 | 24 | 2 | 12.1 | 30 |
| 28425 | FIGS-LR | 6 | IND | 2 | 2 | 30 | 49 | 5.43 | 30 |
| 28556 | FIGS-LR | 6 | TUR | 3 | 3 | 33 | 34 | 3.3 | NA |
| 28613 | FIGS-LR | 6 | TUR | 2 | 2 | 12.1 | 0.2 | 9.03 | 10 |
| 28615 | FIGS-LR | 6 | TUR | 3 | 3 | 8 | 1.2 | 4.8 | NA |
| 28621 | FIGS-LR | 6 | TUR | 2 | 2 | 21 | 5.2 | 2.93 | NA |
| 28624 | FIGS-LR | 2 | TUR | 3 | NA | 18 | 1.1 | 1.65 | NA |
| 28626 | FIGS-LR | 6 | TUR | 1 | 2 | 10 | 0.3 | 4.6 | 16 |
| 28628 | FIGS-LR | 6 | TUR | 2 | 3 | 40 | 0.2 | 0.6 | 30 |
| 28636 | FIGS-LR | 2 | TUR | 2 | 2 | 6 | 1.1 | 4.95 | 5 |
| 28639 | FIGS-LR | 2 | TUR | 2 | 3 | 22.7 | 7 | 0.45 | 6 |
| 28643 | FIGS-LR | 6 | TUR | 2 | 2 | 20 | 1.2 | 1.5 | 12 |
| 28647 | FIGS-LR | 6 | TUR | 3 | 2 | 6.1 | 4.1 | 0.7 | 8 |
| 28648 | FIGS-LR | 6 | TUR | 2 | 2 | 17 | 6.1 | 0.47 | 20 |
| 28674 | FIGS-LR | 6 | TUR | 3 | 3 | 60 | 58 | 10.85 | NA |
| 29088 | FIGS-LR | 6 | JOR | 2 | 2 | 2 | NA | 0.53 | 20 |
| 31545 | FIGS-LR | 6 | JOR | NA | NA | NA | NA | NA | NA |
| 32711 | FIGS-LR | 6 | SYR | 3 | 2 | 11 | NA | 1.4 | 8 |
| 32734 | FIGS-LR | 6 | SYR | NA | NA | NA | NA | NA | NA |
| 33039 | FIGS-LR | 6 | DZA | 2 | 2 | 4.2 | 0.4 | 6.7 | 0.2 |
| 35331 | FIGS-LR | 6 | SYR | 1 | 2 | 4.7 | 0.3 | 7.7 | NA |
| 35542 | FIGS-LR | 6 | SYR | 2 | 3 | 19 | 0.4 | 2.45 | 30 |
| 36497 | FIGS-LR | 2 | ETH | 2 | 2 | 21.5 | 0.6 | 0.83 | NA |
| 36667 | FIGS-LR | 6 | IND | 2 | 3 | 8.2 | NA | 2.63 | 8 |
| 36688 | FIGS-LR | 6 | IND | 2 | 2 | 16 | NA | 3.5 | 40 |
| 36691 | FIGS-LR | 6 | IND | 2 | 2 | 24 | NA | 3.1 | 8 |
| 36714 | FIGS-LR | 6 | IND | 2 | 2 | 2 | NA | 2.07 | 0.2 |
| 36726 | FIGS-LR | 6 | IND | 2 | 3 | 75 | NA | 2.2 | NA |
| 36727 | FIGS-LR | 6 | IND | 3 | 2 | 24 | NA | 4.47 | 12 |
| 36729 | FIGS-LR | 6 | IND | 3 | 2 | 16 | NA | 1 | 20 |
| 36730 | FIGS-LR | 6 | IND | 3 | 4 | 16 | NA | 3.47 | 4 |
| 37541 | FIGS-LR | 6 | LBY | 3 | 3 | 16 | NA | 0.8 | NA |
| 37545 | FIGS-LR | 6 | LBY | 2 | 2 | 32 | NA | 2.47 | NA |
| 38493 | FIGS-LR | 2 | ETH | 2 | 3 | 60 | 16 | 3 | 6 |
| 112497 | FIGS-LR | 2 | GEO | 2 | 3 | 30.2 | 0.4 | 2.5 | 16 |
| 112527 | FIGS-LR | 6 | GRC | 3 | 3 | 16.1 | 3.2 | 8.8 | 12 |
| 112590 | FIGS-LR | 2 | GRC | 2 | 2 | 20.1 | 0.2 | 1.2 | 20 |
| 120512 | FIGS-LR | 6 | GEO | 3 | 2 | 40 | NA | 9.85 | NA |
| 120605 | FIGS-LR | 6 | RUS | 2 | 2 | NA | 2.7 | 40 | 4 |
| 120639 | FIGS-LR | 6 | RUS | 3 | 2 | 12.2 | 40 | 18.7 | 10 |
| 125781 | FIGS-LR | 6 | PSE | 3 | 2 | 8.2 | 0.2 | 15.33 | NA |
| 138196 | FIGS-LR | 2 | ERI | 4 | 4 | 24 | 16 | 26.95 | 27 |
| 143864 | GCP | 6 | TUN | 3 | 1 | 16 | 0.4 | 8.27 | 30 |
| 143867 | GCP | 6 | USA | 3 | 2 | 22 | 5.2 | 4.2 | NA |
| 143872 | GCP | 6 | USA | 4 | 1 | 32 | 0.4 | 4.67 | NA |
| 143873 | GCP | 6 | USA | 3 | 2 | 20 | 0.4 | 4.47 | 16 |
| 143874 | GCP | 6 | USA | 2 | 1 | 5 | 0.2 | 22.7 | 16 |
| 143875 | GCP | 6 | USA | 2 | 1 | NA | 0.7 | 27 | 4 |
| 143876 | GCP | 6 | TUR | 1 | 1 | 16 | 0.8 | 3.73 | NA |
| 143877 | GCP | 6 | TUR | 3 | 2 | 20 | 4.2 | 5.7 | 30 |
| 143878 | GCP | 6 | TUR | 2 | 1 | 37 | 0.2 | 0.6 | 16 |
| 143879 | GCP | 2 | TUR | 4 | 3 | 28 | 7 | 2.3 | 50 |
| 143880 | GCP | 6 | TUR | 3 | 2 | 20 | 1.7 | 0.8 | 10 |
| 143882 | GCP | 6 | ETH | 3 | 2 | 28 | 27 | 3.3 | 16 |
| 143883 | GCP | 6 | GRC | 2 | 1 | 45 | 12.2 | NA | NA |
| 143884 | GCP | 2 | NLD | 2 | 1 | NA | 0.4 | 0.6 | 2 |
| 143886 | GCP | 6 | ETH | 1 | 1 | 50 | 20 | 1.6 | NA |
| 143887 | GCP | 2 | TUR | 4 | 2 | 60 | NA | 5.33 | NA |
| 143888 | GCP | 2 | TUR | 3 | 2 | 40 | 75 | 37.5 | NA |
| 143889 | GCP | 6 | AFG | 2 | 2 | 8 | 33 | 19.5 | 10 |
| 143890 | GCP | 6 | AFG | 3 | 1 | 24 | 8 | 5 | 12 |
| 143893 | GCP | 2 | UKR | 1 | 2 | 24 | 10 | 0.6 | 16 |
| 143894 | GCP | 2 | MKD | 2 | 2 | 26 | NA | 1.4 | 10 |
| 143901 | GCP | 6 | ETH | 2 | 2 | 70 | 10 | 3 | 16 |
| 143905 | GCP | 6 | IND | 3 | 2 | 12 | 30 | 9.33 | NA |
| 143906 | GCP | 2 | JPN | 1 | 1 | 32 | NA | 5 | 16 |
| 143907 | GCP | 6 | JPN | 3 | 2 | 47 | NA | 0.4 | 8 |
| 143909 | GCP | 6 | RUS | 2 | 2 | 69 | 25 | 15 | 2 |
| 143911 | GCP | 2 | ETH | 3 | 2 | 65 | 1 | 0.4 | 2 |
| 143912 | GCP | 6 | ETH | 2 | 2 | 36 | 70 | 4.33 | 16 |
| 143913 | GCP | 6 | ETH | 2 | 2 | 18 | 70 | 5.33 | 3 |
| 143915 | GCP | 6 | ETH | 2 | 3 | 8 | NA | 23 | 30 |
| 143916 | GCP | 6 | ETH | 2 | 2 | 32 | 28 | 3.07 | 20 |
| 143918 | GCP | 6 | ETH | 3 | 2 | 40 | 34 | 4.33 | 16 |
| 143919 | GCP | 6 | ETH | 2 | 2 | 32 | 75 | 5.3 | NA |
| 143921 | GCP | 6 | ETH | 2 | 2 | 24 | 52 | 6.6 | 9 |
| 143923 | GCP | 2 | UKR | 1 | 2 | 40 | 2 | 2.45 | NA |
| 143924 | GCP | 6 | MKD | 2 | 2 | 16 | 20.1 | 7.6 | 4 |
| 143926 | GCP | 6 | DEU | 3 | 2 | 33 | 10 | 0.4 | NA |
| 143927 | GCP | 6 | DEU | 2 | 2 | 16 | 18.2 | 17 | 4 |
| 143929 | GCP | 2 | DEU | 1 | 1 | 14 | 0.4 | 0.2 | 10 |
| 143930 | GCP | 2 | NLD | 2 | 1 | 18 | 0.4 | 4.95 | 20 |
| 143932 | GCP | 2 | MKD | 2 | 2 | 14 | 6 | 12.45 | 24 |
| 143933 | GCP | 6 | MKD | 3 | 2 | 13 | 1.1 | 0.47 | NA |
| 143934 | GCP | 6 | DEU | 3 | NA | 40 | 0.3 | NA | 12 |
| 143936 | GCP | 6 | PAK | 4 | 2 | 16 | 2.2 | 1.7 | 20 |
| 143938 | GCP | 2 | DNK | 1 | 2 | 32 | 4.2 | 0.33 | 18 |
| 143943 | GCP | 6 | ETH | 2 | 2 | 47 | 13 | 2.8 | 16 |
| 143945 | GCP | 6 | DZA | 2 | 2 | 8 | 6.2 | 2.5 | 6 |
| 143949 | GCP | 6 | USA | 2 | 3 | 8 | 0.2 | 0.2 | 18 |
| 143950 | GCP | 6 | USA | 2 | 2 | 22 | 10.2 | 0.6 | 16 |
| 143952 | GCP | 6 | DZA | 2 | NA | 32 | 0.4 | 2.95 | 16 |
| 143953 | GCP | 6 | DZA | 2 | 2 | 24 | 14.5 | 6.05 | 30 |
| 143954 | GCP | 6 | USA | 2 | 2 | 12 | 5.5 | 6.3 | 40 |
| 143955 | GCP | 6 | USA | 2 | 3 | 22 | 1.2 | 8.1 | NA |
| 143958 | GCP | 6 | ETH | 2 | 3 | 40 | 7 | 41.5 | 27 |
| 143963 | GCP | 6 | USA | 1 | 3 | 32 | 31 | 28.95 | 4 |
| 143965 | GCP | 6 | IND | 3 | 3 | 4 | 0.8 | 15.15 | NA |
| 143966 | GCP | 6 | USA | 3 | 4 | 16 | 8.2 | 8.55 | 30 |
| 143967 | GCP | 6 | IND | 2 | 2 | 27 | 2.5 | NA | 9 |
| 143969 | GCP | 6 | ISR | 3 | 2 | 39 | 0.35 | 2.9 | 2 |
| 143971 | GCP | 6 | USA | 3 | NA | 29 | 0.6 | 0.6 | 16 |
| 143973 | GCP | 2 | USA | 2 | 2 | 21 | 2 | 13.6 | 10 |
| 143974 | GCP | 6 | DZA | 3 | 2 | 32 | 0.2 | 32 | NA |
| 143976 | GCP | 6 | DZA | 2 | 1 | 22 | 0.6 | 0.27 | 16 |
| 143977 | GCP | 6 | USA | 2 | 2 | 50 | 0.2 | 4.05 | 16 |
| 143978 | GCP | 6 | EGY | 3 | 1 | 32 | 0.3 | 1.7 | 12 |
| 143980 | GCP | 6 | EGY | 2 | 2 | 24 | 0.4 | 3.2 | 8 |
| 143984 | GCP | 6 | AFG | 3 | 1 | 51 | 0.3 | 1.73 | NA |
| 143986 | GCP | 6 | USA | 2 | 3 | 42 | 0.6 | 6.48 | 12 |
| 143990 | GCP | 6 | AZE | 1 | 2 | 46 | 0.4 | 1.67 | 40 |
| 143991 | GCP | 6 | MAR | 1 | 2 | 43 | 1.1 | 0.87 | NA |
| 143992 | GCP | 6 | USA | 3 | 2 | 25 | 0.5 | 2.2 | NA |
| 143994 | GCP | 6 | USA | 3 | 2 | 56 | 0.4 | 0.8 | NA |
| 143996 | GCP | 6 | USA | 2 | 2 | 43 | 0.2 | 0.4 | 30 |
| 143997 | GCP | 6 | USA | 2 | 2 | 53 | 2.2 | 7.55 | 16 |
| 143998 | GCP | 6 | USA | 1 | 1 | 24 | 0.4 | 1.6 | 4 |
| 143999 | GCP | 6 | USA | 1 | 1 | 62 | 2.1 | 5.3 | NA |
| 144000 | GCP | 6 | AZE | 2 | 2 | 12 | 2 | 6.8 | 4 |
| 144004 | GCP | 6 | USA | 3 | 2 | 32 | 4.2 | 2.4 | 30 |
| 144006 | GCP | 2 | CAN | 3 | 1 | 8 | 0.2 | 0.33 | 4 |
| 144007 | GCP | 6 | USA | 2 | 2 | 27 | 3.2 | 2.5 | NA |
| 144008 | GCP | 6 | USA | 2 | 3 | 25 | 0.25 | 2.9 | NA |
| 144011 | GCP | 2 | USA | 2 | NA | 46 | NA | NA | NA |
| 144013 | GCP | 2 | TUR | 2 | 3 | 70 | 16.4 | 20.2 | 24 |
| 144016 | GCP | 2 | TUR | 4 | 3 | 66 | 10 | 30.25 | 50 |
| 144019 | GCP | 2 | TUR | 2 | 1 | 54 | 0.8 | 14 | 30 |
| 144020 | GCP | 6 | AFG | 2 | 2 | 54 | 60 | 23.67 | 30 |
| 144021 | GCP | 6 | USA | 2 | 2 | 48 | 26 | 6.6 | 16 |
| 144023 | GCP | 2 | RUS | 3 | 2 | 32 | 55 | 10.2 | NA |
| 144024 | GCP | 6 | IRN | 4 | 4 | 45 | 55 | 15.25 | NA |
| 144025 | GCP | 2 | IRN | 2 | 3 | 80 | 10 | 8 | 20 |
| 144029 | GCP | 6 | IRN | 3 | 1 | 52 | NA | 4.87 | 6 |
| 144030 | GCP | 2 | IRN | 2 | 2 | 53 | NA | 4 | NA |
| 144031 | GCP | 6 | CAN | 2 | 2 | 61 | 3 | 0.27 | 10 |
| 144033 | GCP | 6 | USA | 2 | 1 | 75 | 2 | 5.93 | 2 |
| 144034 | GCP | 6 | USA | 2 | 2 | 40 | 2 | 28 | 12 |
| 144035 | GCP | 2 | ETH | 2 | 2 | 62 | NA | 25 | NA |
| 144036 | GCP | 6 | USA | 3 | 2 | 40 | 9.5 | 13.15 | 10 |
| 144039 | GCP | 6 | TUR | 2 | 2 | 85 | 2 | 6.27 | 6 |
| 144040 | GCP | 6 | TUR | 2 | 2 | 52 | 18 | 4.85 | NA |
| 144043 | GCP | 2 | TUR | 2 | 2 | 0.2 | 8 | 4.93 | NA |
| 144045 | GCP | 2 | TUR | 2 | 2 | 26 | 30 | 12.25 | 50 |
| 144046 | GCP | 6 | TUR | 3 | 2 | 26 | 4 | 0.3 | 4 |
| 144047 | GCP | 6 | USA | 2 | 2 | 26 | 13 | 0.6 | 16 |
| 144048 | GCP | 6 | JOR | 1 | 2 | 32 | 20 | 2.33 | NA |
| 144049 | GCP | 2 | JOR | 2 | 2 | 48 | 3 | 0.2 | 16 |
| 144050 | GCP | 6 | JOR | 2 | 3 | 66 | 0.4 | 0.4 | 16 |
| 144056 | GCP | 6 | MAR | 1 | 2 | 20.1 | 2.1 | 0.3 | 8 |
| 144059 | GCP | 6 | MAR | 1 | 2 | 57 | 15.1 | 0.3 | NA |
| 144062 | GCP | 6 | MAR | 3 | 3 | 30 | 1.1 | 1.47 | 6 |
| 144064 | GCP | 6 | MAR | 1 | 1 | 2 | 0.2 | 0.4 | 6 |
| 144068 | GCP | 6 | MAR | 2 | 2 | 36 | 15.2 | NA | 8 |
| 144070 | GCP | 6 | MAR | 1 | 2 | 36 | 4 | 10.6 | 12 |
| 144071 | GCP | 6 | MAR | 1 | 2 | 55 | 0.4 | 7.27 | 30 |
| 144075 | GCP | 6 | CHN | 3 | 2 | 36 | 0.3 | 4.93 | 16 |
| 144076 | GCP | 6 | CHN | 1 | 1 | 24 | NA | 2.13 | 8 |
| 144077 | GCP | 6 | CHN | 3 | 1 | 65 | NA | 9.75 | 16 |
| 144078 | GCP | 2 | CHN | 3 | 2 | 48 | 19.5 | 0.87 | 40 |
| 144079 | GCP | 6 | CHN | 2 | 3 | 77 | 32 | 18 | 20 |
| 144081 | GCP | 6 | CHN | 3 | 2 | 53 | 0.9 | 0.33 | NA |
| 144084 | GCP | 6 | CHN | 2 | 2 | 36 | 8.4 | 11.33 | NA |
| 144085 | GCP | 6 | IRQ | 2 | 1 | 47 | 5.5 | 8.2 | 30 |
| 144088 | GCP | 2 | DZA | 1 | 2 | 36 | 0 | 1.73 | NA |
| 144090 | GCP | 6 | JOR | 3 | 1 | 32 | 1.2 | 0.5 | 8 |
| 144091 | GCP | 2 | JOR | 2 | 2 | 24 | 0.2 | 4 | 8 |
| 144095 | GCP | 2 | JOR | 2 | 2 | 36 | 0.5 | 2.73 | 16 |
| 144097 | GCP | 2 | CZE | 2 | 2 | 41 | 3.5 | 0.27 | 60 |
| 144098 | GCP | 2 | IND | 3 | 2 | 8 | 2.2 | 0.4 | 16 |
| 144100 | GCP | 2 | MKD | 2 | 2 | 16 | 0.4 | 12 | 30 |
| 144104 | GCP | 2 | YEM | 3 | 2 | 61 | 0.6 | 0.5 | 16 |
| 144105 | GCP | 6 | TUN | 3 | 2 | 4.2 | 0.4 | 1.07 | 12 |
| 144107 | GCP | 6 | TUN | 3 | 2 | 32 | 0.3 | 4.4 | 8 |
| 144108 | GCP | 6 | TUN | 1 | 1 | 52 | 1.7 | 16.93 | 16 |
| 144135 | GCP | 6 | IRQ | 2 | 1 | 43 | 7 | 3.53 | 16 |
| 144140 | GCP | 6 | GRC | 2 | 2 | 4.1 | 0.3 | 12.35 | 12 |
| 144141 | GCP | 6 | IRN | 1 | 2 | 37 | 0.6 | 4 | 16 |
| 144144 | GCP | 2 | IRN | 3 | 2 | 16 | NA | 32.67 | 16 |
| 144145 | GCP | 2 | IRN | 2 | 2 | 0.4 | 4 | 24 | 10 |
| 144146 | GCP | 2 | IRN | 4 | 2 | 57 | 37.25 | 41.25 | 12 |
| 144147 | GCP | 6 | IRN | 3 | 2 | 60 | 22.25 | 43.5 | 20 |
| 144148 | GCP | 6 | IRQ | 2 | 1 | 75 | 5.5 | 29.95 | 30 |
| 144150 | GCP | 2 | AFG | 2 | 2 | 60 | NA | 15.2 | NA |
| 144151 | GCP | 6 | EGY | 2 | 1 | 28 |  | 7 | 40 |
